# Genomic characterization of the uncultured *Bacteroidales* family *S24-7* inhabiting the guts of homeothermic animals

**DOI:** 10.1186/s40168-016-0181-2

**Published:** 2016-07-07

**Authors:** Kate L. Ormerod, David L. A. Wood, Nancy Lachner, Shaan L. Gellatly, Joshua N. Daly, Jeremy D. Parsons, Cristiana G. O. Dal’Molin, Robin W. Palfreyman, Lars K. Nielsen, Matthew A. Cooper, Mark Morrison, Philip M. Hansbro, Philip Hugenholtz

**Affiliations:** Australian Centre for Ecogenomics, School of Chemistry and Molecular Biosciences, The University of Queensland, Brisbane, Australia; Priority Research Centre for Healthy Lungs, The University of Newcastle and Hunter Medical Research Institute, Newcastle, Australia; QFAB Bioinformatics, The University of Queensland, Brisbane, Australia; Australian Institute for Bioengineering and Nanotechnology, The University of Queensland, Brisbane, Australia; Institute for Molecular Bioscience, The University of Queensland, Brisbane, Australia; Microbial Biology and Metagenomics, The University of Queensland Diamantina Institute, Translational Research Institute, Brisbane, Australia

**Keywords:** Gut microbiome, S24-7, *Homeothermaceae*, Population genomes, Metagenomics, Comparative genomics

## Abstract

**Background:**

Our view of host-associated microbiota remains incomplete due to the presence of as yet uncultured constituents. The *Bacteroidales* family *S24-7* is a prominent example of one of these groups. Marker gene surveys indicate that members of this family are highly localized to the gastrointestinal tracts of homeothermic animals and are increasingly being recognized as a numerically predominant member of the gut microbiota; however, little is known about the nature of their interactions with the host.

**Results:**

Here, we provide the first whole genome exploration of this family, for which we propose the name “*Candidatus* Homeothermaceae,” using 30 population genomes extracted from fecal samples of four different animal hosts: human, mouse, koala, and guinea pig. We infer the core metabolism of “*Ca.* Homeothermaceae” to be that of fermentative or nanaerobic bacteria, resembling that of related *Bacteroidales* families. In addition, we describe three trophic guilds within the family, plant glycan (hemicellulose and pectin), host glycan, and α-glucan, each broadly defined by increased abundance of enzymes involved in the degradation of particular carbohydrates.

**Conclusions:**

“*Ca.* Homeothermaceae” representatives constitute a substantial component of the murine gut microbiota, as well as being present within the human gut, and this study provides important first insights into the nature of their residency. The presence of trophic guilds within the family indicates the potential for niche partitioning and specific roles for each guild in gut health and dysbiosis.

**Electronic supplementary material:**

The online version of this article (doi:10.1186/s40168-016-0181-2) contains supplementary material, which is available to authorized users.

## Background

The host microbiome has been firmly established as critical to host physiology. Evidence now supports the microbiome as influential in diverse processes ranging from infection susceptibility [1] to behavior [2]. Unique anatomical sites are occupied by microbiota of distinct composition [3], supporting alternative functions being carried out at each site. Of clear significance is the gut microbiome, as metabolic capacity is a product of the capabilities encoded within both the host and the microbiome. The typical vertebrate gut microbiome is dominated by the *Firmicutes* and *Bacteroidetes*, and the divergent nature of gut-associated genera in comparison to other phylum members not associated with this environment indicates host selection and evolution occurring over a long period [4]. The relationship is also dynamic, evidenced by shifts in the composition of the gut microbiota encountered with perturbations to a person’s diet, as well as with many acute and chronic, non-communicable diseases, such as inflammatory bowel diseases, or with their treatment (reviewed in [5, 6]). Despite our advances in describing these fluctuations, many members of the communities have yet to be cultured and characterized. As such, it remains difficult to ascribe their contributions to gut and systemic function, and thereby, host health and well-being.

One such uncharacterized inhabitant of the gastrointestinal tract is a novel branch of the “*Bacteroides* group” first recognized in 2002 [7]. This branch was subsequently classified by Greengenes [8] and Silva [9] as an uncultured family of the order *Bacteroidales*, named after one of the earliest environmental clones belonging to the lineage, S24-7 (acc. AJ400263, [7]). Multiple studies have since reported the altered abundance of *S24-7* family members in association with different environmental conditions, e.g., *S24-7* is more abundant in diabetes-sensitive mice fed a high-fat diet, in particular when chow is supplemented with gluco-oligosaccharides [10] and following treatment-induced remission of colitis in mice [11]. Members of the *S24-7* family are also differentiated by their degree of IgA-labeling [12, 13] suggesting at least some members of the group are targeted by the innate immune system. While these observations are currently limited to murine-based studies, they do suggest that *S24-7* is involved in host-microbe interactions that impact on gut function and health.

To further our understanding of the *S24-7* family, we obtained 30 population genomes from four different hosts (human, mouse, koala, and guinea pig) and performed a comparative genomics analysis. The recovered genomes define a family that is most closely related to, but distinct from, the genera *Barnesiella* and *Coprobacter*. Analysis of 16S rRNA gene databases suggests a strong habitat preference for the homeothermic gut. Metabolically, we infer *S24-7* are fermentative or nanaerobic [14] species, consistent with their environmental niche. We describe three trophic guilds within the family focusing on α-glucan, host glycan, or plant glycan-based carbohydrates suggesting the capacity for niche partitioning and/or divergent spatial organization of its members.

## Results

### “*Candidatus* Homeothermaceae” (*S24-7*) members are found almost exclusively in the guts of homeothermic animals

**Fig. 1:**
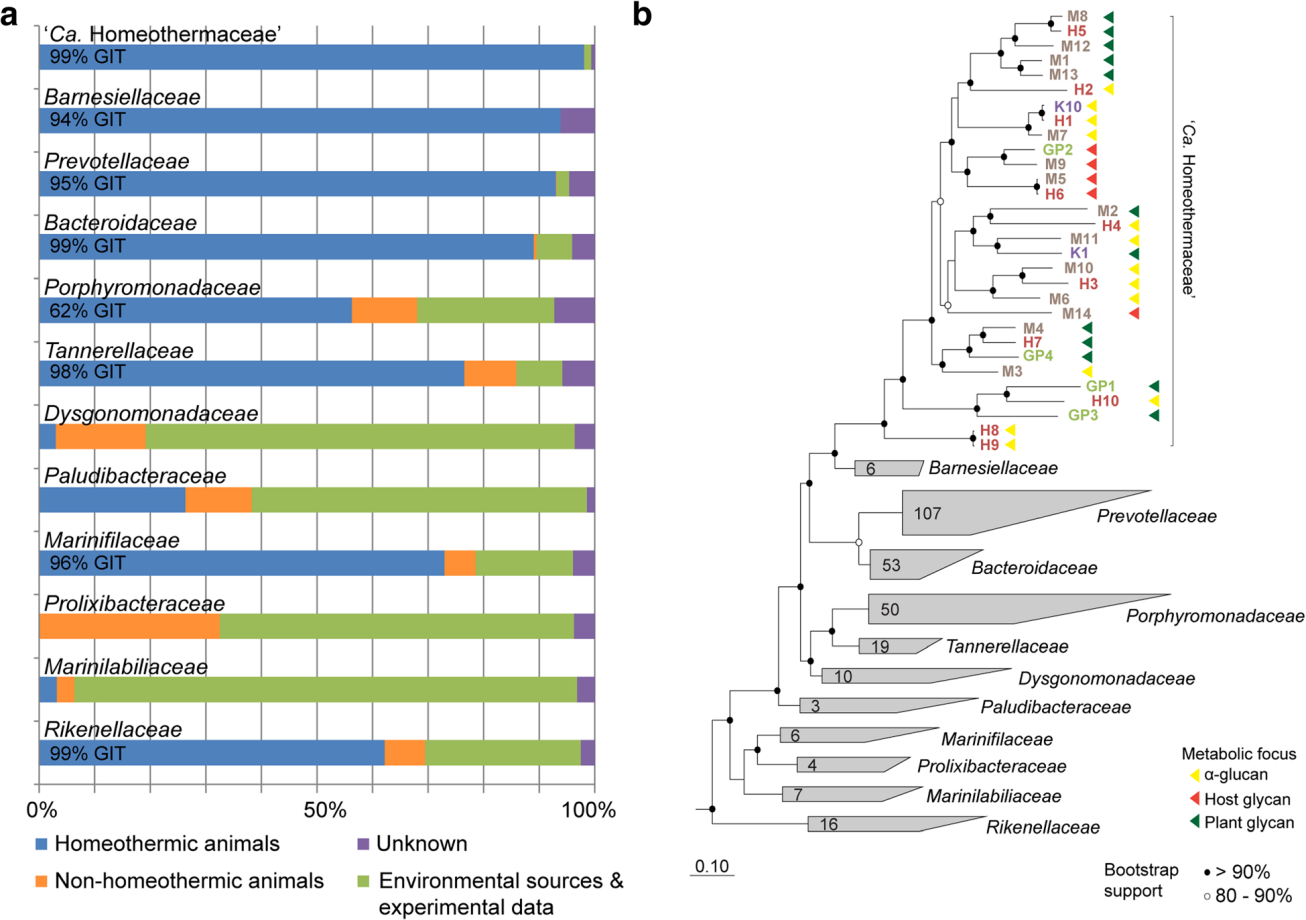
Habitat and phylogenetic positioning of “*Ca.* Homeothermaceae” (*S24-7*). **a** Habitat source of 16S rRNA gene sequences described in the Silva database [9] belonging to members of the *Bacteroidales* order. Written percentages indicate the proportion of homeothermic animal samples which were derived from the gastrointestinal tract (GIT) for families dominated by homeothermic samples. Note that families in this panel have been reclassified according to the genome tree (panel **b**). **b** A maximum-likelihood tree of “*Ca.* Homeothermaceae” population genomes based on alignment of 120 concatenated marker genes. Bootstrap support derived from 100 replicates. Genome identifiers indicate host from which genome was recovered: human (*H*), mouse (*M*), guinea pig (*GP*), or koala (*K*). The metabolic focus, as determined by carbohydrate active gene annotations, is indicated by *colored triangles* following each population genome

The Silva database (release 119, [9]) contains over 3000 sequences designated as being within the *S24-7* family. Notably, 98 % of these sequences originate from homeothermic animals, 99 % of which are associated with the gastrointestinal system (Fig. 1a). There is a single sequence obtained from the intestine of the anchovy *Coilia mystus*. We therefore propose the name “*Candidatus* Homeothermaceae” in reference to the homeothermic preference of the family. Similar, although not as extreme, trends are observed in other *Bacteroidales* families, both in terms of homeothermic hosts and gastrointestinal preference (Fig. 1a). The association of *Bacteroidales* with feces is well documented and has led to the establishment of host-specific detection of fecal contamination based on this order [15, 16]. Of the 57 unique animal species identified as hosting “*Ca.* Homeothermaceae,” the majority (96 %) are herbivores or omnivores, with many of the omnivores likely to consume a mostly herbivorous diet (e.g., chimpanzee, gorilla). The only two carnivorous hosts within the database are the dhole (*Cuon alpinus*), one of only four canids classified as hypercarnivores [17], and the sea lion. The substantial dominance of plant-based diets amongst “*Ca.* Homeothermaceae” hosts potentially reflects the metabolic capacity of the family.

### “*Ca.* Homeothermaceae” population genomes

We obtained 30 near complete “*Ca.* Homeothermaceae” draft genomes from fecal metagenomic datasets: 10 from human (genomes H1 to H10), 14 from mouse (*Mus musculus*, order: *Rodentia*, family: *Muridae*; genomes M1 to M14), four from guinea pig (*Cavia porcellus*, order: *Rodentia*, family: *Caviidae*; genomes GP1 to GP4), and two from koala (*Phascolarctos cereus*, order: *Diprotodontia*, family: *Phascolarctidae*; K1 and K10). Average genome size was 2.69 Mb, with a notable outlier from the koala gut of 4.46 Mb (Additional file 1: Table S1). Abundance of each population bin within their respective metagenomic dataset varied from 0.4 to 14.8 % indicating that members of this family represent large fractions of the gut community in some animal hosts (Additional file 1: Table S1). We selected the most complete genome with the lowest inferred contamination, M4 (99.4 % complete; 0.4 % contamination), as a representative of the “*Ca.* Homeothermaceae” family, for which we propose the name “*Candidatus* Homeothermus arabinoxylanisolvens.” Identification of protein orthologs between each of the genomes revealed a core of 503 proteins present in at least 28 of the 30 assembled “*Ca.* Homeothermaceae” genomes with an average of 14 % unique genes (minimum 6 % (H9), maximum 28 % (K1), Additional file 2: Table S2). Unique genes were distributed throughout each genome, including the large genome obtained from koala, with only a small number clustered in apparent genomic islands (Additional file 3: Figure S1).

### Genome-based phylogenetic classification of the “*Ca.* Homeothermaceae”

The assembled “*Ca.* Homeothermaceae” population genomes were phylogenetically placed within the *Bacteriodales* order by generating a genome tree based on 120 single-copy marker genes within 300 *Bacteroidales* reference genomes obtained from NCBI (Fig. 1b). The closest characterized relatives of “*Ca.* Homeothermaceae” are members of the bacterial genera *Barnesiella* and *Coprobacter*. These genera are currently classified as members of the family *Porphyromonadaceae* but according to our genome-based inference, we propose that they be reclassified as a separate family, *Barnesiellaceae* fam. nov. (Fig. 1b and Additional file 4: Figure S2). Calculation of average nucleotide sequence identity (ANI) between “*Ca.* Homeothermaceae” genomes supports the dataset representing 27 different species, with only three examples of members of the same species having been sampled on two independent occasions (ANI >95 %, [18]). Two of these genome pairs were recovered from different hosts: M5 and H6, originating from mouse and human, and H1 and K10, originating from human and koala (Additional file 5: Figure S3). The third pair, H8 and H9, shares a human origin. Thus, “*Ca.* Homeothermaceae” species are not restricted to specific hosts. Above the species level, there are also five clades within the dataset sharing increased average amino acid identity that may represent distinct genera (Fig. 1b and Additional file 5: Figure S3). Nine population genomes fall outside of both inferred species and genera indicating further diversity exists within the family.

### Shared features of “*Ca.* Homeothermaceae” genomes

**Fig. 2:**
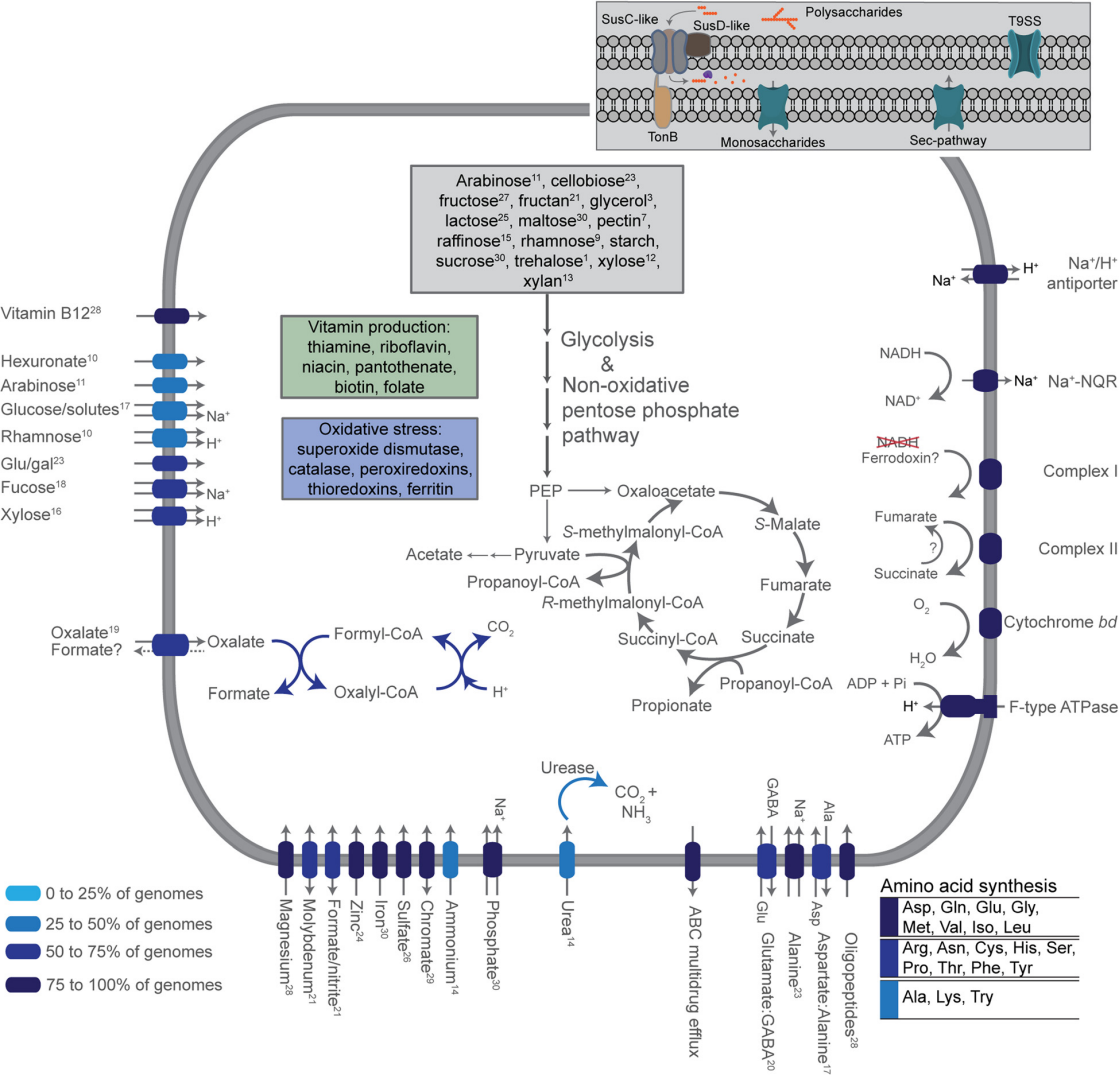
Inferred key metabolism of “*Ca.* Homeothermaceae.” Model constructed using KEGG orthology annotations in addition to manual curation. *Superscript numbers* denote the number of population genomes encoding a given characteristic

Annotation of each genome supports the family as being composed of primary fermenters capable of producing acetate, propionate, and succinate (Fig. 2). The “*Ca.* Homeothermaceae” cell envelope is that of a diderm (Gram-negative), as demonstrated by the absence of typical monoderm protein domains and the presence of the majority of typical diderm domains, consistent with the *Bacteroidetes* phylum (Additional file 6: Table S3, [19]). A number of the genomes encode putative capsular polysaccharide synthesis loci defined by the presence of the regulatory homolog UpxY in association with a number of glycosyltransferase genes, as seen in *Bacteroides* (Additional file 7: Figure S4, [20]). Predicted fimbrial genes are present in 80 % of the genomes and carry the FimA domain (pfam06321), and/or the associated fimbrillin C domain (pfam15495), or, more commonly, the Mfa-like-1 (pfam13149) or Mfa2 (pfam08842) domain indicating “*Ca.* Homeothermaceae,” may produce fimbriae resembling that of the periodontal pathogen *Porphyromonas gingivalis* where they have been shown to bind both host proteins and those of other bacteria, as well as promote inflammatory responses (reviewed in [21]). All “*Ca.* Homeothermaceae” contain at least one putative antibiotic efflux pump, and 60 % also encode a class A β-lactamase (Additional file 7: Figure S4), a profile that is less extensive than that of their close relatives *Bacteroides* (reviewed in [22])*.*

All genomes encode the capacity for the production of vitamins B_1_ (thiamine), B_2_ (riboflavin), B_3_ (niacin), B_5_ (pantothenate), B_7_ (biotin), and B_9_ (folate), a range which is consistent with other *Bacteroidetes* [23]. No homolog of PdxH, the final enzyme required for active vitamin B_6_ production, was identified in any of the genomes, despite the remainder of the pathway being present. The lack of PdxH has also been noted in some *Bacteroides* species [23]. The complete vitamin B_12_ (cobalamin) production pathway is absent from all “*Ca.* Homeothermaceae,” however, a subset encode a partial pathway originating from adenosyl cobyrinic acid. Vitamin B_12_ transporters were identified within 28 of the 30 genomes (Fig. 2). Therefore, members of this family are predicted to rely on neighboring populations for the production of B_12_, an important cofactor, as is seen in other *Bacteroidetes* [24].

In addition to fermentation capacity, “*Ca.* Homeothermaceae” also encode elements of an electron transport chain indicating possible alternative modes of energy production. Complex I is found in the majority of “*Ca.* Homeothermaceae” genomes (25 of 30, Additional file 7: Figure S4) and comprises 11 of the 14 canonical subunits [25], lacking NuoEFG, the NADH dehydrogenase module. Such complexes are found in multiple phyla; however, their redox function is often unclear [26]. Of the remaining five genomes, four (GP3, GP4, M10, and M14) have loci adjacent to contig boundaries suggesting incomplete assembly as the reason for the missing elements. The remaining genome, H5, entirely lacks all components of complex I. While this genome has a relatively low level of completeness (85 %), other genomes with similar completion levels contain complex I, suggesting true absence in H5. The closest relative of this population, M8, contains a complete (11/14 subunit) complex, indicating this would be a recent loss from H5 (Fig. 1b). An F-type ATP synthase was identified in 26 “*Ca.* Homeothermaceae” genomes and is integrated within the genomic locus of complex I. Consequently, several genomes with incomplete complex I also harbor incomplete ATP synthases, including H5 in which all subunits are absent.

In addition to complex I, complex II (fumarate reductase/succinate dehydrogenase) is present in 28 genomes and is composed of three subunits: a flavoprotein, an iron-sulfur protein, and a single transmembrane protein, indicating a type B structure [27]. Both genomes with incomplete complex II gene sets, GP3 (missing two genes) and GP4 (missing all genes), were also missing elements of complex I, consistent with their lower completeness and increased fragmentation (Additional file 7: Figure S4 and Additional file 1: Table S1). The flavoprotein catalytic subunit of the complex resembles that of other related anaerobic *Bacteroidales* species suggesting fumarate as the terminal electron acceptor in an anaerobic respiratory chain, as is described in *Bacteroides* (Additional file 8: Figure S5, [28]). However, also like *Bacteroides* and most other members of the *Bacteroidales* order, the majority of “*Ca.* Homeothermaceae” genomes contain a vertically inherited aerobic reductase operon, *cydAB* (Additional file 9: Figure S6), a high-affinity bd-type oxidase induced under low oxygen conditions permitting growth in nanomolar concentrations of oxygen [29, 30]. Thus, “*Ca.* Homeothermaceae” are likely nanaerobes, able to inhabit both anoxic and marginally oxic environments [14]. Four genomes lack the *cydAB* operon (H4, H9, M9, and GP4), one of which is 97 % complete, suggesting that not all “*Ca.* Homeothermaceae” have this capacity. We were unable to confirm the operons’ absence through genomic context due to a lack of synteny in the surrounding regions despite close relatives in some instances (H8-H9, M9-GP2). If truly absent, as with complex I in H5, this would indicate relatively recent independent loss of this operon from multiple “*Ca.* Homeothermaceae” lineages. The variable presence of respiratory complexes in “*Ca.* Homeothermaceae” suggests a level of energetic flexibility within the family and potentially relatively recent purging of non-essential respiratory elements in the gastrointestinal environment.

In addition to the typical electron transport chain elements, the Na^+^ translocating NADH:ubiquinone oxidoreductase Nqr complex was identified in 27 “*Ca.* Homeothermaceae” genomes (Additional file 7: Figure S4). Nqr permits the use of a Na^+^ gradient for energy production and suggests NADH may be oxidized by this complex in “*Ca.* Homeothermaceae” rather than by complex I, which is missing the NADH dehydrogenase module. All six Nqr subunits are present within the 27 genomes, which include H5. Three genomes, H2, H9, and M11, are missing all six components. At least one H^+^/Na^+^ antiporter is found within all “*Ca.* Homeothermaceae” genomes supporting the use of this system.

To support a prediction of “*Ca.* Homeothermaceae” as nanaerobes, we looked for proteins within the genomes that would provide oxidative stress protection. Superoxide dismutase (O_2_^−^ to O_2_ or H_2_O_2_) was identified in 24 of the genomes, and eight of these also encode a catalase (H_2_O_2_ to H_2_O + O_2_) protein (Additional file 7: Figure S4). All eight catalase-positive genomes were obtained from mice. Peroxide reduction may also be achieved via several peroxiredoxins that are present within the “*Ca.* Homeothermaceae” genomes. Firstly, the alkyl hydroperoxide reductase, AhpC, and associated disulfide reductase, AhpF, were identified in nine genomes. A further six contained AhpC only, representing a separate cluster within generated gene trees (Additional file 7: Figure S4 and Additional file 10: Figure S7). There are also between one and four copies of rubrerythrin in all “*Ca.* Homeothermaceae” genomes. Finally, 25 genomes encode the thiol peroxidase bacterioferritin comigratory protein. Protein stability and reduced state regeneration during oxidative stress is supported by the presence of between two and six TRX family (group I) thioredoxins within each genome and a single copy of the thioredoxin reductase TrxB in all but three genomes: GP1 and M13 lack TrxB, while M12 contains two copies. In addition, 26 genomes contain a non-heme ferritin protein permitting the storage of excess iron. Overall, “*Ca.* Homeothermaceae” members appear well equipped to deal with oxidative stress, supporting potential microaerobic growth.

### The “*Ca.* Homeothermaceae” secretome

Proteins secreted by gut-inhabiting bacteria can influence interactions with both the host and other microbes. Approximately 15 % of “*Ca.* Homeothermaceae” proteins carry the general secretory pathway signal peptide, as predicted by SignalP [31], which is at the lower end of the range predicted for Gram-negative species (13 to 42 %) [32]. Within this secretome, ~15 % of the proteins were annotated with carbohydrate-based activity and thus potentially provide nutrients for “*Ca.* Homeothermaceae” or neighboring populations. Several immune-related peptidases are also secreted: 20 of the 30 population genomes contain a metalloprotease belonging to peptidase family M6 (immune inhibitor A family) (Additional file 7: Figure S4). Members of this family have been demonstrated to degrade antimicrobial peptides [33] and components of the extracellular matrix [34] and may therefore play a role in invasiveness or persistence within the host (reviewed in [35]). In addition, 11 genomes contain an IgA degrading peptidase (peptidase family M64) that may assist with immune evasion by these populations [36].

Working in concert with the general secretory pathway, a type IX secretion system (T9SS) was also identified in the majority of the “*Ca.* Homeothermaceae” genomes. All 10 components of the system (PorK, PorL, PorM, PorN, PorP, PorT, PorU, PorV, PorW, and Sov) were present in 22 genomes (other genomes contained incomplete gene sets), in addition to the regulatory two-component sensor system, PorX (response regulator) and PorY (histidine kinase), responsible for the co-regulation of a subset of T9SS genes (Additional file 7: Figure S4, [37]). No other secretion system was identified within “*Ca.* Homeothermaceae”. Within the T9SS, PorU acts as a peptidase for proteins containing a conserved C-terminal domain (TIGR04183) that dictates the use of the system and is cleaved during translocation [38, 39]. We identified 161 proteins containing this domain within the “*Ca.* Homeothermaceae” genomes, ~75 % of which also carried a general secretory pathway signal peptide, supporting their movement to the periplasm and subsequent secretion by the T9SS. The majority of proteins within this group are annotated as hypothetical (60 %); however, there is a homolog of a characterized immune-related peptidase, streptopain (SpeB). SpeB, encoded by *Streptococcus pyogenes*, contains the peptidase C10 domain and is capable of degrading multiple components of the immune system (reviewed in [40]). Streptopain homologs are present in 26 “*Ca.* Homeothermaceae” genomes.

### Potential metabolic guilds within “*Ca.* Homeothermaceae”

**Fig. 3:**
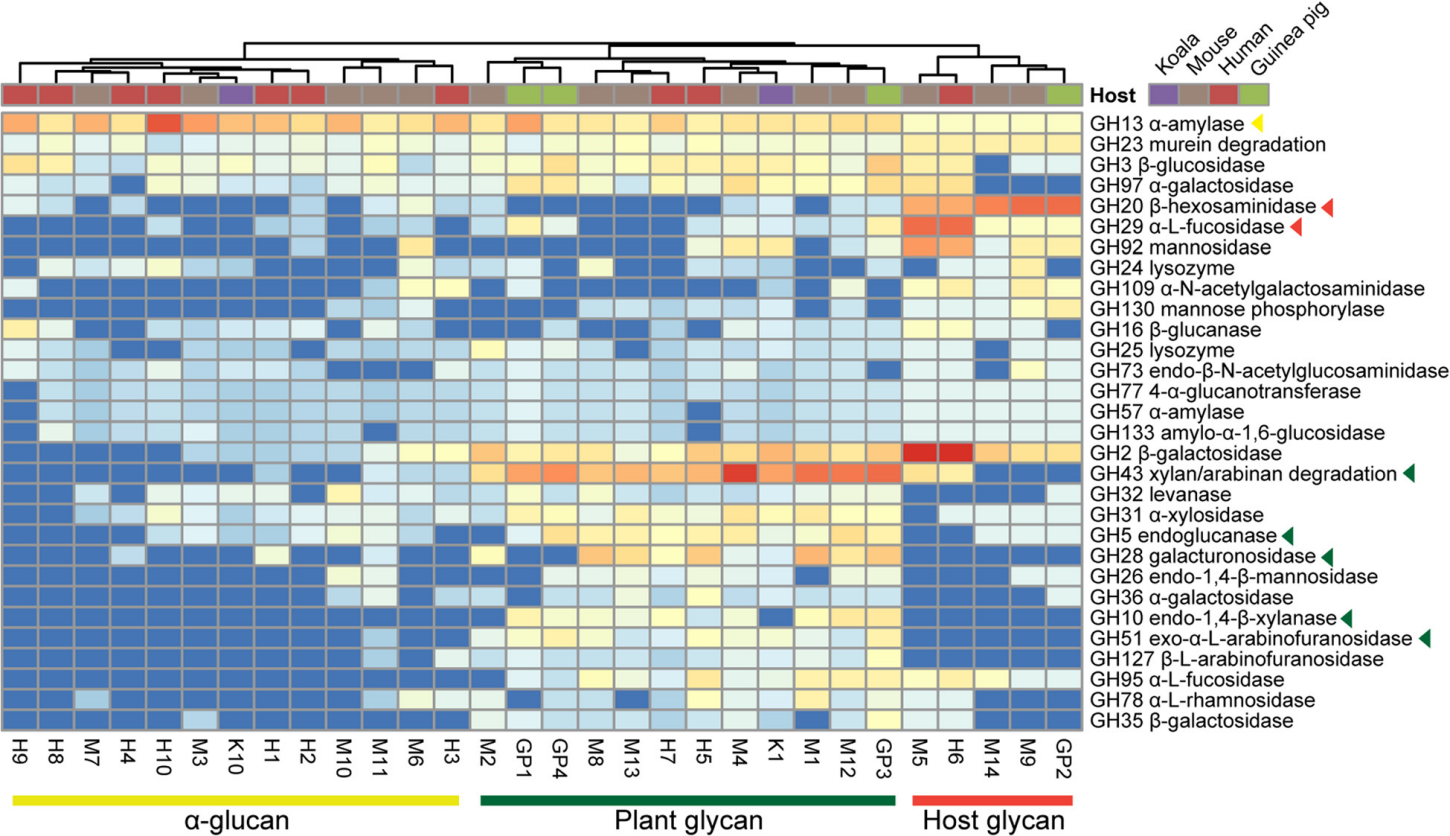
Carbohydrate use differs amongst “*Ca.* Homeothermaceae” population genomes. Heatmap displaying the 30 most abundant glycoside hydrolase categories within “*Ca.* Homeothermaceae” population genomes. Colored lines denote predicted carbohydrate focus. Matching colored triangles indicate categories significantly enriched within a particular guild in comparison to both alternative guilds (Additional file 13: Table S6)

Carbohydrate-active enzymes constitute ~6 % of “*Ca.* Homeothermaceae” coding sequences, a level similar to that of other *Bacteroidales* families (Additional file 11: Table S4). GH13 is the most abundant glycoside hydrolase family and largely comprises ɑ-amylases, suggesting starch is a key resource of the family. In support of this, GH13 was found to be significantly (*P* = 1.52E-08) enriched in “*Ca.* Homeothermaceae” in comparison to related *Bacteroidales* families, as was the starch binding module CBM26 (*P* = 1.37E-06, Additional file 12: Table S5). The next most abundant glycoside hydrolase family is GH43 and is dominated by xylosidase and arabinosidase enzymes capable of degrading xylan and arabinan, respectively. However, this ability is not ubiquitous amongst “*Ca.* Homeothermaceae” as 12 genomes contain no genes in this category (Fig. 3). Differential abundance of such enzymes indicates potential niche partitioning, and comparative analysis across the population genomes suggests three trophic guilds with differential capacity for carbohydrate degradation: α-glucans, complex plant cell wall glycans (hemicellulose and pectin), or host-derived glycans (Figs. 1b and 3).

The α-glucan and plant glycan guilds constitute the majority of the “*Ca.* Homeothermaceae” genomes, comprising 13 and 12 genomes respectively, with the host glycan group composed of five members. We used a combination of indicator species identification and pairwise differential abundance analysis to confirm the enriched enzymes within each group, retaining those enzymes identified by both methods as defining the guild (Additional files 13 and 14: Tables S6 and S7). The α-glucan guild is the most highly selective group, with only two significantly enriched enzyme categories, both starch related (Additional file 13: Table S6). The plant guild is equipped for the degradation of arabinan, xylan, and pectin, all plant cell wall constituents. Finally, the host glycan guild is enriched in β-hexosaminidases, capable of cleaving glucosamine and galactosamine residues, α-fucosidases, capable of cleaving fucose residues and comprises the only genomes to contain the sialic acid cleaving sialidase, supporting a capacity for host glycan degradation (other enzymes carrying the GH33 domain do not display homology to known sialidase enzymes, Additional file 15: Figure S8). Integration of trophic guild membership with phylogeny reveals the presence of some clades with a shared substrate focus, while others are mixed (Fig. 1b). Each guild is also mixed in terms of host distribution, with no foci found in only one host. There are, however, dominant guilds within both guinea pig and human samples: 70 % of human origin genomes are α-glucan focused and 75 % of guinea pig origin genomes are plant focused. This may reflect diet preference of these hosts; however, more genomes are required to provide support for this theory. We also predict that these guilds may occupy distinct spatial niches within the gut; the host glycan group primarily associating with the mucus layer, as has been demonstrated for the known mucin degrader *Akkermansia muciniphila* [41], and the plant and starch groups associating primarily with the digesta.

**Fig. 4:**
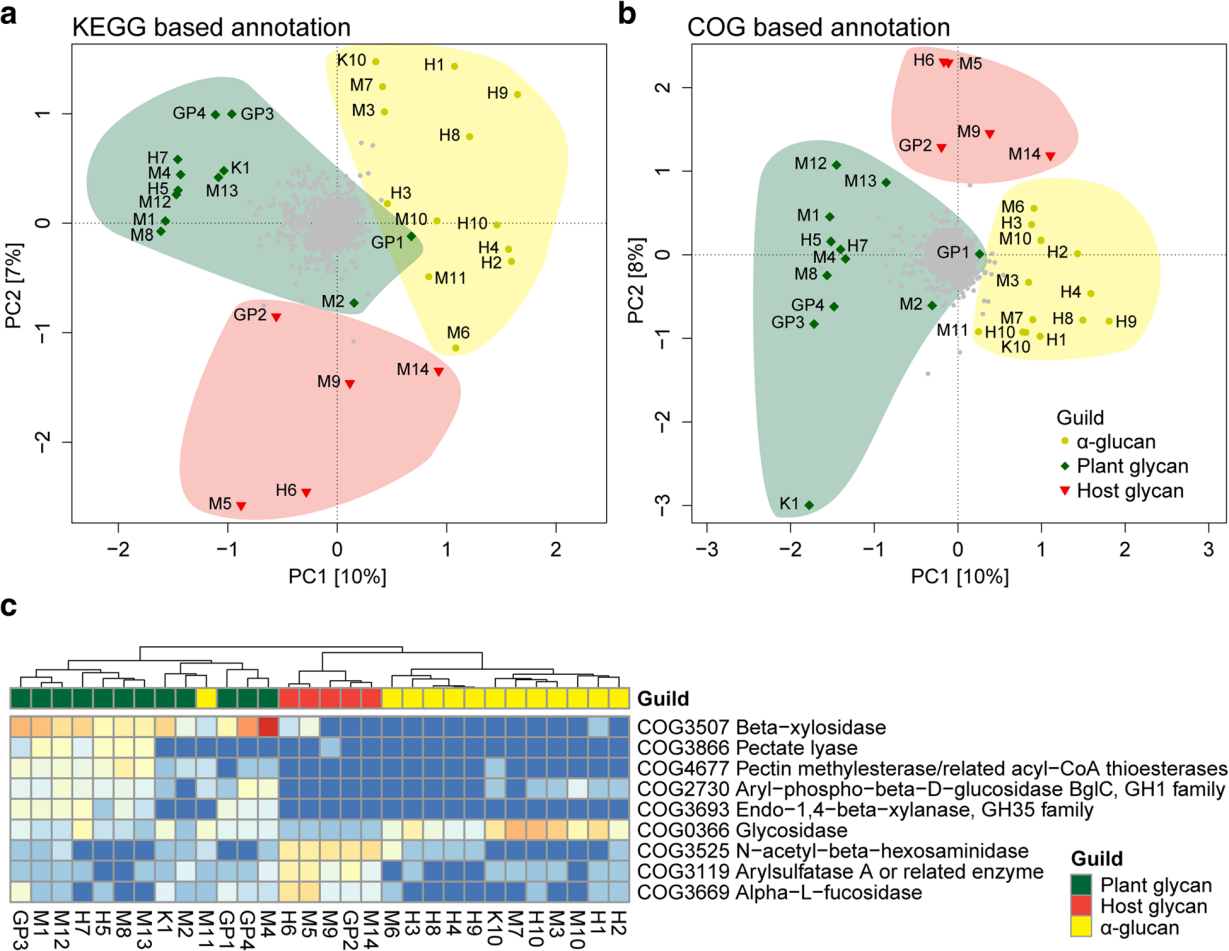
Metabolic guilds cluster together based on broad KEGG and COG orthology annotation. **a** PCA plot generated using KEGG annotation of “*Ca.* Homeothermaceae” population genomes. **b** PCA plot generated using COG annotation of “*Ca.* Homeothermaceae” population genomes. **c** COGs enriched in pairwise comparisons for each trophic guild (Additional file 17: Table S9)

To provide contextual data, we generated carbohydrate-active enzyme (CAZy) profiles for a selection of microbial isolates from the gut and compared their glycoside hydrolase distribution with that of the “*Ca.* Homeothermaceae” population genomes (Additional file 15: Figure S8). The trophic guild division between “*Ca.* Homeothermaceae” genomes remained largely intact with this extended analysis; however, two genomes from the α-glucan guild, M6 and M11, became associated with members of an alternate guild, M6 with host glycan and M11 with plant glycan. Both these populations are positioned on the boundary between their two alternative guilds when broader analysis was conducted based on Clusters of Orthologous Groups (COG) and Kyoto Encyclopedia of Genes and Genomes (KEGG) orthology annotation (Fig. 4a, b). In support of the predicted metabolic guilds, *A. muciniphila*, a known mucin degrader [42], was found to associate with the host glycan guild (Additional file 15: Figure S8). The plant guild associates with a single species, *Prevotella copri*, which has been demonstrated to increase in abundance following a controlled diet containing barley kernel-based bread [43]. Functional metagenomic analysis of fecal samples following this controlled diet revealed an increase in genes necessary for the degradation of polysaccharides within the bread, including xylan-degrading enzymes which were found to be enriched within the plant guild (Additional file 13: Table S6). Finally, the α-glucan guild associates with the acetogen *Blautia hydrogenotrophica*, which is known to harbor few glycoside hydrolases and consequently has been proposed to inhabit a syntrophic niche within the gut [28]. Therefore, the α-glucan guild may actually represent populations utilizing the metabolism of their neighbors and providing an alternative function in return.

### Polysaccharide utilization loci

Carbohydrate degrading enzymes may be clustered together in polysaccharide utilization loci (PUL), first described in *Bacteroides thetaiotaomicron* [20] and now known to be typical of *Bacteroidetes* [44]. Such loci are defined by the presence of orthologs of the starch utilization system (Sus) components responsible for starch and maltooligosaccharide binding (SusD) and transfer to the periplasmic space (SusC) [45, 46]. Multiple *susCD*-like gene pairs are found within all “*Ca.* Homeothermaceae” genomes, and most also contain the gene pair in association with carbohydrate-active enzymes (Additional file 16: Table S8). In addition, ~10 % of “*Ca.* Homeothermaceae” PULs are located in close proximity to a hybrid two-component system protein, which have been demonstrated to regulate PUL expression (Additional file 16: Table S8) [47, 48]. While homologs of extracytoplasmic function σ-factors, also linked to PUL expression [49], were identified in many “*Ca.* Homeothermaceae” genomes, they were not located near PULs. The average number of both *susCD* pairs and pairs with associated carbohydrate enzymes is highest in the plant guild, suggesting these as requiring the most varied carbohydrate degradative machinery (Additional file 16: Table S8). Both *susC* and *susD* are present in large numbers throughout the *Bacteroidetes* with variable sequence identity existing both within and between species [20, 50, 51]. While initially described as a starch binding system, the association of *susCD* pairs with enzymes targeting a variety of substrates supports the broader use of this system, and the differential regulation of distinct *susCD* pairs in response to dietary changes has been demonstrated in *B. thetaiotaomicron* [52]. We constructed gene trees based on both genes (data not shown) to determine whether there was greater variability within a particular “*Ca.* Homeothermaceae” guild, which could reflect an ability to bind and transport a wider variety of substrates. However, average phylogenetic diversity scores [53] for all three guilds were similar for both *susC* (α-glucan:1.6, host glycan: 1.7, plant:1.5) and *susD* (α-glucan:1.8, host glycan:1.8, plant:1.7) suggesting that equivalent sequence diversity exists within each group and therefore potentially a similar, although not necessarily overlapping, diversity of substrates available to this system.

### Broader functional comparative analysis of “*Ca.* Homeothermaceae”

To determine whether the metabolic guilds extend to broader genome content, we annotated each genome using both KEGG and COG orthology detection. Ordination plots generated from both annotation systems clustered the metabolic guilds discretely, although with low levels of separation (Fig. 4a, b). Only a single KEGG orthology group was found to be significantly enriched; K12373 (hexosaminidase), which was increased within the host glycan guild, consistent with the previous CAZy-based observations. COG annotation yielded several enriched protein families within each guild (Fig. 4c and Additional file 17: Table S9), all of which are associated with carbohydrate-active enzymes, indicating these as the key differentiating characteristic of each group. In addition to functions noted previously, the host glycan guild is also enriched for arylsulfatase-related enzymes (COG3119), which plays a role in the degradation of host glycans and is therefore consistent with the guild focus.

Using COG-based annotations, we then extended the analysis to compare “*Ca.* Homeothermaceae” to other *Bacteroidales* families. “*Ca.* Homeothermaceae,” *Bacteroidaceae*, and *Prevotellaceae* separated clearly based on COG annotations, while *Porphyromonadaceae* members were intermingled with other families, potentially reflecting the phenotypic diversity of this family (Additional file 18: Figure S9, [54]). The abundance of over 450 individual COGs was found to be significantly different within “*Ca.* Homeothermaceae” compared to the other families; ~75 % of which were decreased (Additional file 19: Table S10). Out of those with increased abundance, several are of interest. Multiple urease-associated COGs were significantly enriched, and the urease gene cluster was subsequently identified in twelve genomes, suggesting a role in nitrogen recycling and a source of ammonia (Additional file 7: Figure S4). Gene trees produced for each of the urease subunits confirm that the presence of urease is unusual within the *Bacteroidales* and suggest lateral transfer of the gene cluster to the common ancestor of “*Ca.* Homeothermaceae” from an *Alistipes*- or *Odoribacter*-like ancestor and subsequent loss from a subset of the “*Ca.* Homeothermaceae” genomes (Additional file 20: Figure S10, data not shown). Putative formyl-CoA transferases (COG1804) were also enriched in “*Ca.* Homeothermaceae,” revealing the presence of the oxalate degrading gene pair formyl-CoA transferase and oxalyl-CoA decarboxylase in 19 of the 30 genomes (Additional file 7: Figure S4). Oxalate is likely transported into the cell via a permease located adjacent to oxalyl-CoA decarboxylase in all 19 oxalate degraders (and which is not found in “*Ca.* Homeothermaceae” lacking the oxalate degrading gene pair) as suggested by genomic analysis of other oxalate degrading species [55]. Oxalate degradation appears to be linked to metabolic guilds, that is, 10 of 12 plant, and four of five host glycan-focused “*Ca.* Homeothermaceae” have oxalate degrading genes, whereas only five of the 13 α-glucan-focused guild have them. As with urease, gene trees confirm that this function is rare within the *Bacteroidales* (Additional file 21: Figure S11, data not shown).

### Relative abundance and prevalence within the sampled mammalian hosts

A large portion of “*Ca.* Homeothermaceae” sequences within 16S rRNA databases originate from mice suggesting high prevalence of the family in the murine host. To determine whether this reflects true prevalence or database bias, we searched for evidence of “*Ca.* Homeothermaceae” in available gut metagenome datasets from both mice and humans. We found “*Ca.* Homeothermaceae,” as represented by the analyzed population genomes, present in ~50 % of mice samples, with an average relative abundance of 6 % of the gut community as estimated by read mapping (from ~100 metagenomes, Additional file 22: Table S11). The prevalence in humans was lower, ~20 %, with an average abundance of ~2 % of the community (from ~300 metagenomes). “*Ca.* Homeothermaceae” is therefore more common in mice than humans but is nonetheless likely to be present in a sizable fraction of the human population.

The most prevalent “*Ca.* Homeothermaceae” species was H8/H9 in 20 % of human datasets, and M6 in 35 % of murine datasets (Additional file 22: Table S11), both members of the α-glucan guild. We were interested to see if this prevalence was consistent across different dietary backgrounds as a potential source of support for our proposed guild structure. To do this, we subdivided the analyzed datasets according to available diet-related metadata. We found mice fed a high-fat diet carried a narrower range of “*Ca.* Homeothermaceae” populations, and these were present at a lower abundance than those fed a standard chow diet (Additional file 22: Table S11). However, no particular guild showed dominance, and M6 remained the most prevalent population overall. Within the public human datasets sampled, very few non-α-glucan guild representatives were detected (Additional file 22: Table S11) in agreement with the dominance of this guild within the “*Ca.* Homeothermaceae” genomes recovered from humans and suggestive of the human diet as most supportive of members of the α-glucan guild. We found a higher prevalence of “*Ca.* Homeothermaceae” in obese individuals (23 %) than in lean (10 %), suggesting that a higher energy diet may better support the family, although there was no dietary composition information available for this dataset. We also identified a substantial increase in the prevalence of “*Ca.* Homeothermaceae” within the Hadza hunter-gatherer population; 70 % of individuals were found to carry at least one population. Only two species were identified within this group of individuals: the typically prevalent H8/H9 and species H4, also a member of the α-glucan guild. The Hadza diet is heavily plant based, composed of tubers, meat, honey, foliage, and berries, with tubers being particularly important due to their constant availability [56]. Tubers consumed by the Hadza contain a large portion of indigestible fibers that are expectorated after chewing. As such, tubers provide a source of moisture, simple sugars, starch, and soluble fiber [56]. The increased prevalence of “*Ca.* Homeothermaceae” within the Hadza suggests this diet is particularly amenable to the maintenance of the identified species, although does not result in any increase in their overall abundance.

## Discussion

The *Bacteroidales* family *S24-7* is encountered frequently in culture-independent studies and is gaining recognition due to both its prevalence, particularly in murine-based datasets, and its fluctuating abundance in cross-sectional and intervention type studies. Increased abundance has been described in mice fed a low-fat diet and, in association with increased exercise [57], in diabetes-sensitive mice fed a high-fat diet [10] and following remission of colitis in a mouse model [11]. *S24-7* is also the dominant family during hibernation of arctic ground squirrels [58]. The consequence of these fluctuations in the abundance of *S24-7* is currently unknown, as they remain uncultured and no genomic studies have been undertaken. Here, we recover a set of *S24-7* population genomes from metagenomic samples, enabling inference of their core metabolism, and propose the name “*Ca.* Homeothermaceae” reflecting an ecological distribution limited to the guts of homeothermic animals (Fig. 1a).

Carbohydrate composition and availability is known to be a primary driver of microbial community structure in gut ecosystems [48, 59–61]. We identified three trophic guilds within the “*Ca.* Homeothermaceae” based on their encoded carbohydrate-active enzymes (Fig. 3) that suggest the family has the capacity to occupy multiple niches within the gut, which may include spatial partitioning. Similar metabolic differentiation is observed in other gut-inhabiting genera such as *Bacteroides* [48, 50, 62] and *Bifidobacterium* [61] where different species encode alternative carbohydrate utilization machinery. Some gut inhabitants may be able to occupy multiple carbohydrate-based trophic niches: *B. thetaiotaomicron* displays a preference for diet-derived polysaccharides, such as xylan-, pectin-, and arabinose-based compounds, over host glycans; however, when such polysaccharides are scarce, host glycan degradation activity is upregulated [52]. “*Ca.* Homeothermaceae” guild representatives may also utilize this strategy; all analyzed population genomes encode starch-degrading ɑ-amylases and as such, while they may have a preference for their specialist carbohydrate source, starch can serve as a foundation carbohydrate for all family members.

We identified two characteristics within a subset of “*Ca.* Homeothermaceae” populations that were relatively unusual for members of the *Bacteriodales*: the capacity for oxalate degradation and the production of urease (Additional files 20 and 21: Figure S10 and S11). Plants are the primary source of dietary oxalate, which in excess contributes to the formation of renal stones by complexing calcium (reviewed in [63]). In agreement with this dietary source, the majority of “*Ca.* Homeothermaceae” populations within the plant-focused guild encode the necessary components for oxalate degradation. Oxalate degrading potential is also encoded within four of the five host glycan guild population genomes while only 40 % of α-glucan guild members contain the necessary genes (Additional file 7: Figure S4). *Oxalobacter formigenes* is the best characterized oxalate degrading gut bacterium, and colonization is associated with the decreased incidence of calcium oxalate stone formation [64]. While *O. formigenes* is dependent on the presence of oxalate and uses it as a sole carbon source [65], other oxalate degraders, such as lactic acid bacteria, require the presence of an additional carbon source [55, 66]. “*Ca.* Homeothermaceae” oxalate degraders are likely in the latter category due to sporadic distribution of the trait across the family. This distribution appears to be the result of lineage-specific loss rather than multiple independent lateral acquisitions (Additional file 20: Figure S10). Urease releases ammonia from urea, which can then be incorporated into microbial amino acids [67] and contributes to nitrogen level stability of the host particularly when protein consumption is low [68]. Urease activity can therefore be advantageous for both host and microbe. However, urease-positive microbes can be detrimental in combination with elevated circulating ammonia levels associated with liver disease [69]. Urease is also a recognized virulence factor in both bacterial and fungal infection (reviewed in [70]). The abundance of urease genes within the gut microbiota in humans differs with age and geography and is potentially reflective of diet [71]. We identified urease positive “*Ca.* Homeothermaceae” populations in all four hosts (Additional file 7: Figure S4), and presence did not correlate with a particular trophic guild making it difficult to predict a role for urease within the group; however, a metabolic role for the enzyme is supported by the presence of glutamine synthetase in the majority of genomes (Fig. 2).

A key question relating to newly characterized members of the microbiota is whether they are friend or foe. “*Ca.* Homeothermaceae” representatives have been shown to be IgA coated [12]. IgA production is induced by both commensal and pathogenic intestinal inhabitants, and both are believed to be able to induce the production of highly specific IgA leading to microbial cell coating [72]. “*Ca.* Homeothermaceae” are found in both IgA+ and IgA− fractions of fecal microbiota [12, 13], however, on the whole, are not highly coated with IgA in contrast to families known to be inflammatory in murine models of colitis, such as *Prevotellaceae* [12]. The presence within some “*Ca.* Homeothermaceae” genomes of an IgA protease (Additional file 7: Figure S4) may contribute to their identification within both IgA+ and IgA− community fractions, although the significance of IgA proteases in vivo remains unclear (reviewed in [73]). The majority of “*Ca.* Homeothermaceae” genomes also contain a homolog of SpeB (Additional file 7: Figure S4), a peptidase capable of degrading multiple immunologically relevant proteins (reviewed in [40]). SpeB homologs are found in other gut bacteria including *Bacteroides fragilis* and *B. thetaiotaomicron* [74, 75] and in the periodontal pathogen *Prevotella intermedia* where the homolog interpain A is involved in the inhibition of the immune response via complement degradation [76]. The presence of multiple potential immune evasion peptidases within members of “*Ca.* Homeothermaceae” does not preclude a typically commensal relationship with the host; however, they may provide the capacity for opportunistic infection under the appropriate conditions [75].

## Conclusions

Overall, this study provides the first genomic insights into the uncultured gut-inhabiting “*Ca.* Homeothermaceae” family through the comparative analysis of 30 draft genomes obtained from metagenomic datasets. We describe varied carbohydrate utilization mechanisms existing within the family in agreement with other genera occupying the same environmental niche. As a group that is particularly prevalent within a key experimental environment, the mouse gut (and also present in the human gut and potentially relevant to human health), further reports confirming the roles of “*Ca.* Homeothermaceae” in vivo are likely to appear in the future.

### Description of “*Candidatus* Homeothermus”

*Homeothermus* (Ho.me.o.ther’mus Gr. adj. *homoios*, similar, Gr. n. *thermē*, heat. *Homeothermus* of homeothermic origin). Inferred to be Gram-negative, non-motile, nanaerobic, and able to ferment a wide range of carbohydrates including arabinose, cellobiose, fructan, fructose, glycerol, lactose, maltose, raffinose, sucrose, xylan, and xylose, with a focus on arabinan and xylan based on enzyme abundance.

### Description of “*Ca.* Homeothermus arabinoxylanisolvens”

*Homeothermus arabinoxylanisolvens* (Ho.me.o.ther’mus Gr. adj. *homoios*, similar, Gr. n. *thermē*, heat. a.rab.in.o.xy.lan.i.sol’vens n. *arabin*, a carbohydrate derived from gum arabic, M.L. n. *xylan*, xylan, M. L. part. adj. *solvere*, to loosen, untie, free up). Description is the same as that for genus “*Ca.* Homeothermus.” Represented by population genome M4 (acc. no. LUJO00000000) obtained from metagenomic sequencing of *Mus musculus* fecal sample.

### Description of “*Ca.* Homeothermaceae”

The description is the same as for the genus “*Ca.* Homeothermus” with the following additions; *-aceae* ending to denote a family. Additional fermentation substrates include pectin, rhamnose, and trehalose. Three trophic guilds are proposed within the family based on the relative abundance of carbohydrate-active enzymes with different substrates: α-glucans, complex plant cell wall components, and host glycans. Type genus: “*Ca.* Homeothermus.” Order: *Bacteroidales*.

### Emended description of the family *Porphyromonadaceae* Krieg, Staley et al. 2011

The description is the same as that given by Krieg et al. [54] with the following amendment. The genera, *Barnesiella*, *Butyricimonas*, *Coprobacter*, *Dysgonomonas*, *Odoribacter*, *Paludibacter*, *Parabacteroides*, *Proteiniphilum*, “*Ca.* Sanguibacteroides,” and *Tannerella* have been removed as they do not form a monophyletic group with the type genus, *Porphyromonas.*

### Description of *Barnesiellaceae* fam. nov.

Includes the genera *Barnesiella* (type genus) and *Coprobacter*. Description is drawn from that of *Barnesiella* given by Sakamoto et al. [77] and *Coprobacter* given by Shkoporov et al. [78]: *-aceae* ending to denote a family. Cells are Gram-negative, obligately anaerobic, non-spore-forming, and non-motile. Saccharolytic. Type genus: *Barnesiella*. Order: *Bacteroidales*.

### Description of *Dysgonamonadaceae* fam. nov.

Includes the genera *Dysgonomonas* (type genus) and *Proteiniphilum*. Description is drawn from that of *Dysgonomonas* given by Hofstad et al. [79] and *Proteiniphilum* given by Chen et al. [80]: *-aceae* ending to denote a family. Cells are Gram-negative, fermentative, and facultatively (*Dysgonomonas*) or obligately (*Proteiniphilum*) anaerobic. Type genus: *Dysgonomonas*. Order: *Bacteroidales*.

### Emended description of the family *Marinifilaceae* Iino, Mori et al. 2014

The description is drawn from that of *Marinifilaceae* given by Iino et al. [81] with the following amendment. The family *Marinifilaceae* contains the genera *Butyricimonas*, *Marinifilum* (type genus), *Odoribacter*, and “*Ca.* Sanguibacteroides.” Cells are Gram-negative, non-spore-forming, non-motile, and facultatively (*Marinifilum*) or obligately (*Butyricimonas*, *Odoribacter*) anaerobic.

### Description of *Paludibacteraceae* fam. nov.

Includes the genus *Paludibacter* (type genus). Description is the same as for the genus *Paludibacter* given by Ueki et al. [82]: *-aceae* ending to denote a family. Type genus: *Paludibacter*. Order: *Bacteroidales*.

### Description of *Tannerellaceae* fam. nov.

Includes the genera *Parabacteroides* and *Tannerella* (type genus). Description is drawn from that of *Tannerella* given by Sakamoto et al. [83] and *Parabacteroides* given by Sakamoto et al. [84]: *-aceae* ending to denote a family. Cells are Gram-negative, non-motile, and obligately anaerobic. Type genus: *Tannerella*. Order: *Bacteroidales*.

## Methods

### Sample collection and sequencing

Fecal samples were obtained from six 12-week-old female C57BL/6 mice housed in accordance with the University of Newcastle Animal Care and Ethics Committee; reference number A-2013-303. DNA was extracted from feces using the PowerSoil DNA Isolation Kit (MO BIO Laboratories, CA, USA) according to the manufacturer’s instructions. Library preparation was performed using the Nextera DNA Library Preparation Kit (Illumina, CA, USA). Libraries were sequenced at the Diamantina Institute, The University of Queensland, using the Illumina HiSeq 2500 platform generating ~9 Gbp of 100 bp paired-end reads per sample.

Koala fecal samples originated from a 12-year-old male as previously described [85]. Public metagenomic datasets generated from fecal samples of both human and guinea pig were downloaded from the NCBI sequence read archive (SRA); human samples were obtained from multiple projects, specifically runs (ERR209459, ERR209707, ERR525737, ERR688517, ERR688528, ERR710427, ERR710429, SRR413598, SRR413599); guinea pig samples were obtained from a single project (BioProject: SRP012966).

### Sequence assembly and population genome recovery

Reads from mouse fecal samples were adapter trimmed and merged using SeqPrep (https://github.com/jstjohn/SeqPrep) then remaining pairs were quality trimmed using Nesoni v0.128 (https://github.com/Victorian-Bioinformatics-Consortium/nesoni) with a minimum Phred quality threshold of 20. Assembly of pooled reads was performed using CLC Genomics Workbench v7.0.4 (QIAGEN, Aarhus, Denmark) using a word size of 30 and bubble size of 1500. Scaffolding was performed during assembly, and reads were mapped back to contigs with default settings. Minimum contig length was 300. Mapping of reads to final assemblies was performed using BWA v0.7.10 [86] with default settings.

SRA data from guinea pig and human datasets was quality and adapter trimmed using Trimmomatic v0.3.2 [87] with default settings plus a head crop of 10 and minimum read length of 30. Trimmed reads were merged using BBMerge (https://sourceforge.net/projects/bbmap/). Assembly was performed either per individual run (human) or pooled (guinea pig) using CLC Genomics Workbench v8.5.1 (QIAGEN, Aarhus, Denmark) either using default settings (human) or using a word size of 30 and a bubble size of 1000 (guinea pig). Minimum contig length was 500. Scaffolding was performed during assembly, and reads were mapped back to contigs with default settings. Gap filling was performed on assemblies from human datasets using Abyss-sealer [88] with default settings. Mapping of reads to final assemblies was performed using BamM v1.5.1 (http://ecogenomics.github.io/BamM/) with default settings.

Population genomes were obtained either from previous studies [85] or de novo from metagenomic datasets using GroopM v0.2 ([89], mouse) or MetaBAT v0.25.4 ([90], human, guinea pig). Phylogenetic analysis and estimation of contamination and completeness of recovered genomes was performed using CheckM v1.0.3 [91], which utilizes a set of single-copy marker genes. “*Ca.* Homeothermaceae” genomes were refined by removing contigs with incongruent coverage profiles as identified by RefineM v0.0.3 (https://github.com/dparks1134/RefineM). Gap filling was performed on these assemblies using FinishM v0.0.6 (https://github.com/wwood/finishm). Reads mapping to each genome were extracted from a complete assembly mapping (incorporating refined genomes and additional unrefined genomes as the reference) using BamM v1.5.1 (http://ecogenomics.github.io/BamM/) then remapped to each individual genome using CLC Genomics Workbench v8.5.1 (QIAGEN, Aarhus, Denmark) with default settings. These mappings were used for manual investigation of specific genomic features.

### Phylogenetic resolution

A maximum-likelihood tree of “*Ca.* Homeothermaceae” population genomes in the context of the order *Bacteroidales* was generated based on an alignment of 120 concatenated single-copy, bacterial-specific, marker genes implemented within an in-house pipeline. Marker genes from 300 *Bacteroidales* and 37 *Sphingobacteriales* (outgroup) genomes were obtained from publicly available genomes within the NCBI database and aligned using Mafft v7.221 [92]. A maximum-likelihood tree was inferred using FastTree v2.1.7 [93] under the WAG + GAMMA model based on alignment positions containing a residue within ≥90 % of sequences (36,713 positions). Bootstrap support was derived from 100 replicates.

Individual gene trees were generated using FastTree v2.1.7 [93] implemented within Mingle v0.0.15 (https://github.com/Ecogenomics/mingle) utilizing the BLAST [94] workflow, identifying homologs within the IMG database [95]. Default Mingle settings used for all genes except for oxalyl-CoA decarboxylase where percent identity was increased to 40 % due to an alignment length of <100 amino acids with default settings. Bootstrap support was derived from 100 replicates, also implemented within Mingle. Phylogenetic diversity scores for *susC* and *susD* gene trees were calculated using the picante R package [96]. All trees were visually inspected using ARB v6.0.2 [97].

### Genome annotation and core metabolic analysis

Genomes were annotated using Prokka v1.11 [98]. All subsequent gene-based analysis was performed using the output of this annotation process. Orthologous proteins within each genome were identified using Proteinortho v5.11 [99]. Average nucleotide identity was calculated using the Goris method [18] implemented in calculate_ani.py (available at: https://github.com/widdowquinn/scripts/blob/master/bioinformatics/calculate_ani.py). Average amino acid identity between each genome pair was calculated using CompareM v0.0.5 (https://github.com/dparks1134/CompareM). Protein families were identified using pfam_scan.pl against the Pfam database release 28 [100] employing HMMER v3.1b2 [101]. Archetypal cell envelope families as per Albertsen et al. [19] were used for predicting cell structure. TIGRFAMs were identified using hmmscan from HMMER v3.1b2 [101] against database release 15.0 (downloaded from ftp://ftp.jcvi.org/pub/data/TIGRFAMs/). Antibiotic resistance genes were predicted using the Resfams database [102].

Kyoto Encyclopedia of Genes and Genomes (KEGG) term annotation was performed using KAAS [103] and KEGG maps in combination with RAST [104], KBase (http://kbase.us/), and Pathway Tools v19.0 [105], and curated gene lists [106] were used to elucidate the general metabolic profile of “*Ca.* Homeothermaceae.” Clusters of Orthologous Groups (COG) profiles were identified using BLASTP v2.2.30+ [94] against 2014 update of the 2003 COG database downloaded from NCBI (http://www.ncbi.nlm.nih.gov/COG/, last accessed July 2015) with e-value cutoff of 1e-6.

Carbohydrate-active enzymes (CAZy) were identified using hmmscan from HMMER v3.1b2 [101] against the dbCAN database v4 [107]. Percentage abundance of each CAZy category was assessed against the total number of genes with CAZy annotation within each genome. Signal peptide sequences within CAZy annotated genes were predicted using SignalP v4.1 [31] and LipoP v1.0 [108] with a margin cutoff of 4 for LipoP. Prediction of membrane positioning was based on the presence of a transmembrane domain (SignalP) or a type II signal peptide (LipoP).

### Differential abundance comparisons

Differential abundance of annotated features (COG, KO, CAZy) was analyzed using DESeq2 R package [109] based on count data. Heatmaps were generated using pheatmap [110] following variance stabilizing normalization of data by DESeq2. Indicator annotations were identified using the labdsv R package [111]. PCA plots were created using the vegan R package [112].

### Prevalence and relative abundance

Prevalence of each population genome and overall abundance of “*Ca.* Homeothermaceae” were assessed by mapping public human and mouse gut metagenome datasets downloaded from the SRA against all genomes using BWA v0.7.12 [86]. Genome coverage amounting to ≥0.5 % of total reads was used as the minimum cutoff for the presence of a given population in a particular sample. Cutoff was based on minimum coverage within read mappings of datasets used to produce “*Ca.* Homeothermaceae” population bins and thus represents a conservative estimate. Relative abundance of each population was based on the percentage of total reads attributed to each genome exceeding the minimum cutoff percentage, normalized for genome size. Diet-related datasets were PRJEB7759 (mouse), PRJNA278393 (Hadza), and PRJEB2054 (lean vs. obese). Additional human samples originated from projects PRJEB1220, PRJEB4410, PRJEB6456, and PRJEB7774

## Abbreviations

ANI, average nucleotide identity; AR, antibiotic resistance; CAZy, carbohydrate-active enzyme; COG, Clusters of Orthologous Groups; KEGG, Kyoto Encyclopedia of Genes and Genomes; PUL, polysaccharide utilization loci; Sus, starch utilization system.

## Additional files

Additional file 1: Table S1. “*Ca.* Homeothermaceae” population genome properties. (DOCX 19 kb) Additional file 2: Table S2. Unique genes within each “*Ca.* Homeothermaceae” population genome. (DOCX 14 kb) Additional file 3: Figure S1. Distribution of unique genes in selected “*Ca.* Homeothermaceae” population genomes. Coding sequences within each genome are denoted by vertical yellow lines, unique genes are denoted by vertical blue lines. Classification of unique genes is based on failure to find an ortholog to a given gene in any of the other “*Ca.* Homeothermaceae” genomes using Proteinortho [99]. (TIF 249 kb) Additional file 4: Figure S2. Phylogenetic tree of the order *Bacteroidales*. Maximum-likelihood tree of the *Bacteroidales* based on the concatenated alignment of 120 concatenated marker genes (36,713 amino acids) using genomes available within the NCBI database. Bootstrap support derived from 100 replicates. (TIF 281 kb) Additional file 5: Figure S3. Average nucleotide and amino acid identity between “*Ca.* Homeothermaceae” population genomes. ANI calculated using Goris method [18] implemented in calculate_ani.py (https://github.com/widdowquinn/scripts/blob/master/bioinformatics/calculate_ani.py). AAI calculated using CompareM v0.0.5 (https://github.com/dparks1134/CompareM). Values over 95 % indicate population genomes originating from the same species. (TIF 2541 kb) Additional file 6: Table S3. Cell wall associated PFAMs identified in “*Ca.* Homeothermaceae” genomes. (XLSX 13 kb) Additional file 7: Figure S4. Presence of described characteristics within “*Ca.* Homeothermaceae” population genomes. Presence determined via BLAST [94] search of annotated proteins within each population genome. AR: antibiotic resistance. (TIF 724 kb) Additional file 8: Figure S5. Gene tree of *frdA*, catalytic subunit of complex II. Maximum-likelihood gene tree was inferred using FastTree 2 [93] based on a 640 amino acid alignment of sequences, implemented within the in-house script Mingle (https://github.com/Ecogenomics/mingle). Bootstrap values represent result of 100 replicates. “*Ca.* Homeothermaceae” *frdA* genes shown in blue. Where shown, tips display IMG genome ID, species name and gene annotation. (TIF 475 kb) Additional file 9: Figure S6. Gene tree of *cydA*, cytochrome *bd* subunit. Maximum-likelihood gene tree was inferred using FastTree 2 [93] based on a 500 amino acid alignment of sequences, implemented within the in-house script Mingle (https://github.com/Ecogenomics/mingle). Bootstrap values represent result of 100 replicates. “*Ca.* Homeothermaceae” *cydA* genes shown in blue. Where shown, tips display IMG genome ID, species name and gene annotation. (TIF 347 kb) Additional file 10: Figure S7. Gene tree of *ahpC*, alkyl hydroperoxide. Maximum-likelihood gene tree was inferred using FastTree 2 [93] based on a 180 amino acid alignment of sequences implemented, within the in-house script Mingle (https://github.com/Ecogenomics/mingle). Bootstrap values represent result of 100 replicates. “*Ca.* Homeothermaceae” *ahpC* genes shown in blue. Where shown, tips display IMG genome ID, species name and gene annotation. (TIF 787 kb) Additional file 11: Table S4. Top 20 most abundant CAZy categories within “*Ca.* Homeothermaceae” as a percentage of total genes with CAZy annotation in each genome. (DOCX 17 kb) Additional file 12: Table S5. CAZy categories with significantly different abundance in “*Ca.* Homeothermaceae” in comparison with collated counts from *Bacteroidacea*e, *Prevotellaceae*, and *Porphyromonadaceae. (DOCX 17 kb)*
Additional file 13: Table S6. Significantly enriched carbohydrate-active enzymes within each trophic guild. (DOCX 18 kb) Additional file 14: Table S7. Indicator carbohydrate-active enzymes identified within each trophic guild. (DOCX 23 kb) Additional file 15: Figure S8. 50 most abundant glycoside hydrolases in “*Ca.* Homeothermaceae” and selected gut-associated species. Heatmap displaying the top 50 most abundant GH enzymes within “Ca. Homeothermaceae” in addition to a selection of reference genomes available on NCBI also originating from fecal samples. (TIF 1691 kb) Additional file 16: Table S8.
*SusCD*-like genes present in “*Ca.* Homeothermaceae” genomes. (DOCX 17 kb) Additional file 17: Table S9. Significantly enriched COGs within each trophic guild. (DOCX 15 kb) Additional file 18: Figure S9. COG category abundance between “*Ca.* Homeothermaceae” and related *Bacteroidales* families*.* PCA plots generated using annotated COGs within “*Ca.* Homeothermaceae” and IMG genomes [95] from other families. Family designation of each genome is based on IMG phylogenetic annotation except for *Barnesiellaceae*, which includes *Barnesiella* and *Coprobacter* (previously *Porphyromonadaceae*). (TIF 550 kb) Additional file 19: Table S10. COGs with significantly altered abundance in “*Ca.* Homeothermaceae” versus related *Bacteroidales* families. (XLSX 57 kb) Additional file 20: Figure S10. Gene tree of *ureA*, urease subunit. Maximum-likelihood gene tree was inferred using FastTree 2 [93] based on a 100 amino acid alignment of sequences, implemented within the in-house script Mingle (https://github.com/Ecogenomics/mingle). Bootstrap values represent result of 100 replicates. “*Ca.* Homeothermaceae” *ureA* genes shown in blue. Where shown, tips display IMG genome ID, species name and gene annotation. (TIF 470 kb) Additional file 21: Figure S11. Gene tree of *oxa,* oxalyl-CoA decarboxylase. Maximum-likelihood gene tree was inferred using FastTree 2 [93] based on a 580 amino acid alignment of sequences, implemented within the in-house script Mingle (https://github.com/Ecogenomics/mingle). Bootstrap values represent result of 100 replicates. “*Ca.* Homeothermaceae” *oxa* genes shown in blue. Where shown, tips display IMG genome ID, species name and gene annotation. (TIF 296 kb) Additional file 22: Table S11. Prevalence and relative abundance of “*Ca.* Homeothermaceae” populations within public metagenomics datasets from human and mouse gut. (DOCX 25 kb)

## References

[1] Sekirov I, Tam NM, Jogova M, Robertson ML, Li YL, Lupp C (2008). Antibiotic-induced perturbations of the intestinal microbiota alter host susceptibility to enteric infection. Infect Immun.

[2] Cryan JF, O’Mahony SM (2011). The microbiome-gut-brain axis: from bowel to behavior. Neurogastroenterol Motil.

[3] Cho I, Blaser MJ (2012). The human microbiome: at the interface of health and disease. Nat Rev Genet.

[4] Bäckhed F, Ley RE, Sonnenburg JL, Peterson DA, Gordon JI (2005). Host-bacterial mutualism in the human intestine. Science.

[5] Wlodarska M, Kostic Aleksandar D, Xavier Ramnik J (2015). An integrative view of microbiome-host interactions in inflammatory bowel diseases. Cell Host Microbe.

[6] Spor A, Koren O, Ley R (2011). Unravelling the effects of the environment and host genotype on the gut microbiome. Nat Rev Microbiol.

[7] Salzman NH, de Jong H, Paterson Y, Harmsen HJ, Welling GW, Bos NA (2002). Analysis of 16S libraries of mouse gastrointestinal microflora reveals a large new group of mouse intestinal bacteria. Microbiology.

[8] McDonald D, Price MN, Goodrich J, Nawrocki EP, DeSantis TZ, Probst A (2012). An improved Greengenes taxonomy with explicit ranks for ecological and evolutionary analyses of bacteria and archaea. ISME J.

[9] Quast C, Pruesse E, Yilmaz P, Gerken J, Schweer T, Yarza P (2013). The SILVA ribosomal RNA gene database project: improved data processing and web-based tools. Nucleic Acids Res.

[10] Serino M, Luche E, Gres S, Baylac A, Bergé M, Cenac C (2012). Metabolic adaptation to a high-fat diet is associated with a change in the gut microbiota. Gut.

[11] Rooks MG, Veiga P, Wardwell-Scott LH, Tickle T, Segata N, Michaud M (2014). Gut microbiome composition and function in experimental colitis during active disease and treatment-induced remission. ISME J.

[12] Palm Noah W, de Zoete Marcel R, Cullen Thomas W, Barry Natasha A, Stefanowski J, Hao L (2014). Immunoglobulin A coating identifies colitogenic bacteria in inflammatory bowel disease. Cell.

[13] Bunker Jeffrey J, Flynn Theodore M, Koval Jason C, Shaw Dustin G, Meisel M, McDonald Benjamin D (2015). Innate and adaptive humoral responses coat distinct commensal bacteria with immunoglobulin A. Immunity.

[14] Morris RL, Schmidt TM (2013). Shallow breathing: bacterial life at low O_2_. Nat Rev Microbiol.

[15] Dick LK, Bernhard AE, Brodeur TJ, Domingo JWS, Simpson JM, Walters SP (2005). Host distributions of uncultivated fecal *Bacteroidales* bacteria reveal genetic markers for fecal source identification. Appl Environ Microbiol.

[16] Layton A, McKay L, Williams D, Garrett V, Gentry R, Sayler G (2006). Development of *Bacteroides* 16S rRNA gene TaqMan-based real-time PCR assays for estimation of total, human, and bovine fecal pollution in water. Appl Environ Microbiol.

[17] Van Valkenburgh B (1991). Iterative evolution of hypercarnivory in canids (*Mammalia*: *Carnivora*): evolutionary interactions among sympatric predators. Paleobiology.

[18] Goris J, Konstantinidis KT, Klappenbach JA, Coenye T, Vandamme P, Tiedje JM (2007). DNA-DNA hybridization values and their relationship to whole-genome sequence similarities. Int J Syst Evol Microbiol.

[19] Albertsen M, Hugenholtz P, Skarshewski A, Nielsen KL, Tyson GW, Nielsen PH (2013). Genome sequences of rare, uncultured bacteria obtained by differential coverage binning of multiple metagenomes. Nat Biotechnol.

[20] Xu J, Bjursell MK, Himrod J, Deng S, Carmichael LK, Chiang HC (2003). A genomic view of the human-*Bacteroides thetaiotaomicron* symbiosis. Science.

[21] Yoshimura F, Murakami Y, Nishikawa K, Hasegawa Y, Kawaminami S (2009). Surface components of *Porphyromonas gingivalis*. J Periodontal Res.

[22] Wexler HM (2007). *Bacteroides*: the good, the bad, and the nitty-gritty. Clin Microbiol Rev.

[23] Magnúsdóttir S, Ravcheev DA, de Crécy-Lagard V, Thiele I. Systematic genome assessment of B-vitamin biosynthesis suggests co-operation among gut microbes. Front Genet*.* 2015;6(148). doi:10.3389/fgene.2015.00148. 10.3389/fgene.2015.00148 PMC4403557 25941533

[24] Goodman AL, McNulty NP, Zhao Y, Leip D, Mitra RD, Lozupone CA (2009). Identifying genetic determinants needed to establish a human gut symbiont in its habitat. Cell Host Microbe.

[25] Friedrich T, Scheide D (2000). The respiratory complex I of bacteria, archaea and eukarya and its module common with membrane-bound multisubunit hydrogenases. FEBS Lett.

[26] Moparthi VK, Hagerhall C (2011). The evolution of respiratory chain complex I from a smaller last common ancestor consisting of 11 protein subunits. J Mol Evol.

[27] Lemos RS, Fernandes AS, Pereira MM, Gomes CM, Teixeira M (2002). Quinol:fumarate oxidoreductases and succinate:quinone oxidoreductases: phylogenetic relationships, metal centres and membrane attachment. Biochim Biophys Acta.

[28] Fischbach MA, Sonnenburg JL (2011). Eating for two: how metabolism establishes interspecies interactions in the gut. Cell Host Microbe.

[29] Baughn AD, Malamy MH (2004). The strict anaerobe *Bacteroides fragilis* grows in and benefits from nanomolar concentrations of oxygen. Nature.

[30] Borisov VB, Gennis RB, Hemp J, Verkhovsky MI (2011). The cytochrome *bd* respiratory oxygen reductases. Biochim Biophys Acta.

[31] Petersen TN, Brunak S, von Heijne G, Nielsen H (2011). SignalP 4.0: discriminating signal peptides from transmembrane regions. Nat Methods.

[32] Song C, Kumar A, Saleh M (2009). Bioinformatic comparison of bacterial secretomes. Genomics Proteomics Bioinformatics.

[33] Dalhammar G, Steiner H (1984). Characterization of inhibitor A, a protease from *Bacillus thuringiensis* which degrades attacins and cecropins, two classes of antibacterial proteins in insects. Eur J Biochem.

[34] Vaitkevicius K, Rompikuntal PK, Lindmark B, Vaitkevicius R, Song T, Wai SN (2008). The metalloprotease PrtV from *Vibrio cholerae*: purification and properties. FEBS J.

[35] Singh B, Fleury C, Jalalvand F, Riesbeck K (2012). Human pathogens utilize host extracellular matrix proteins laminin and collagen for adhesion and invasion of the host. FEMS Microbiol Rev.

[36] Kosowska K, Reinholdt J, Rasmussen LK, Sabat A, Potempa J, Kilian M (2002). The *Clostridium ramosum* IgA proteinase represents a novel type of metalloendopeptidase. J Biol Chem.

[37] Nakayama K (2015). *Porphyromonas gingivalis* and related bacteria: from colonial pigmentation to the type IX secretion system and gliding motility. J Periodontal Res.

[38] Seers CA, Slakeski N, Veith PD, Nikolof T, Chen YY, Dashper SG (2006). The RgpB C-terminal domain has a role in attachment of RgpB to the outer membrane and belongs to a novel C-terminal-domain family found in *Porphyromonas gingivalis*. J Bacteriol.

[39] Glew MD, Veith PD, Peng B, Chen YY, Gorasia DG, Yang Q (2012). PG0026 is the C-terminal signal peptidase of a novel secretion system of *Porphyromonas gingivalis*. J Biol Chem.

[40] Nelson DC, Garbe J, Collin M (2011). Cysteine proteinase SpeB from *Streptococcus pyogenes* - a potent modifier of immunologically important host and bacterial proteins. Biol Chem.

[41] Berry D, Stecher B, Schintlmeister A, Reichert J, Brugiroux S, Wild B (2013). Host-compound foraging by intestinal microbiota revealed by single-cell stable isotope probing. Proc Natl Acad Sci.

[42] Derrien M, Vaughan EE, Plugge CM, de Vos WM (2004). *Akkermansia muciniphila* gen. nov., sp. nov., a human intestinal mucin-degrading bacterium. Int J Syst Evol Microbiol.

[43] Kovatcheva-Datchary P, Nilsson A, Akrami R, Lee Ying S, De Vadder F, Arora T (2015). Dietary fiber-induced improvement in glucose metabolism is associated with increased abundance of *Prevotella*. Cell Metab.

[44] Martens EC, Koropatkin NM, Smith TJ, Gordon JI (2009). Complex glycan catabolism by the human gut microbiota: the Bacteroidetes Sus-like paradigm. J Biol Chem.

[45] Reeves AR, D’Elia JN, Frias J, Salyers AA (1996). A *Bacteroides thetaiotaomicron* outer membrane protein that is essential for utilization of maltooligosaccharides and starch. J Bacteriol.

[46] Reeves AR, Wang GR, Salyers AA (1997). Characterization of four outer membrane proteins that play a role in utilization of starch by *Bacteroides thetaiotaomicron*. J Bacteriol.

[47] Sonnenburg ED, Sonnenburg JL, Manchester JK, Hansen EE, Chiang HC, Gordon JI (2006). A hybrid two-component system protein of a prominent human gut symbiont couples glycan sensing in vivo to carbohydrate metabolism. Proc Natl Acad Sci.

[48] Sonnenburg ED, Zheng H, Joglekar P, Higginbottom SK, Firbank SJ, Bolam DN (2010). Specificity of polysaccharide use in intestinal *Bacteroides* species determines diet-induced microbiota alterations. Cell.

[49] Martens EC, Chiang HC, Gordon JI (2008). Mucosal glycan foraging enhances fitness and transmission of a saccharolytic human gut bacterial symbiont. Cell Host Microbe.

[50] Xu J, Mahowald MA, Ley RE, Lozupone CA, Hamady M, Martens EC (2007). Evolution of symbiotic bacteria in the distal human intestine. PLoS Biol.

[51] Zhu A, Sunagawa S, Mende DR, Bork P (2015). Inter-individual differences in the gene content of human gut bacterial species. Genome Biol.

[52] Sonnenburg JL, Xu J, Leip DD, Chen C-H, Westover BP, Weatherford J (2005). Glycan foraging *in vivo* by an intestine-adapted bacterial symbiont. Science.

[53] Faith DP (1992). Conservation evaluation and phylogenetic diversity. Biol Conserv.

[54] Krieg NR, Staley JT, Brown DR, Hedlund BP, Paster BJ, Ward NL (2011). Bergey’s manual of systematic bacteriology, 2 edn.

[55] Turroni S, Bendazzoli C, Dipalo SCF, Candela M, Vitali B, Gotti R (2010). Oxalate-degrading activity in *Bifidobacterium animalis* subsp. *lactis*: impact of acidic conditions on the transcriptional levels of the oxalyl coenzyme A (CoA) decarboxylase and formyl-CoA transferase genes. Appl Environ Microbiol.

[56] Schnorr SL, Candela M, Rampelli S, Centanni M, Consolandi C, Basaglia G et al. Gut microbiome of the Hadza hunter-gatherers. Nat Commun*.* 2014;5. doi:10.1038/ncomms4654. 10.1038/ncomms4654 PMC3996546 24736369

[57] Evans CC, LePard KJ, Kwak JW, Stancukas MC, Laskowski S, Dougherty J (2014). Exercise prevents weight gain and alters the gut microbiota in a mouse model of high fat diet-induced obesity. PLoS One.

[58] Stevenson TJ, Duddleston KN, Buck CL (2014). Effects of season and host physiological state on the diversity, density, and activity of the arctic ground squirrel cecal microbiota. Appl Environ Microbiol.

[59] Kolida S, Meyer D, Gibson GR (2007). A double-blind placebo-controlled study to establish the bifidogenic dose of inulin in healthy humans. Eur J Clin Nutr.

[60] Cantarel BL, Lombard V, Henrissat B (2012). Complex carbohydrate utilization by the healthy human microbiome. PLoS One.

[61] Milani C, Andrea Lugli G, Duranti S, Turroni F, Mancabelli L, Ferrario C (2015). Bifidobacteria exhibit social behavior through carbohydrate resource sharing in the gut. Sci Rep.

[62] Larsbrink J, Rogers TE, Hemsworth GR, McKee LS, Tauzin AS, Spadiut O (2014). A discrete genetic locus confers xyloglucan metabolism in select human gut *Bacteroidetes*. Nature.

[63] Whiteside SA, Razvi H, Dave S, Reid G, Burton JP (2015). The microbiome of the urinary tract: a role beyond infection. Nat Rev Urol.

[64] Troxel SA, Sidhu H, Kaul P, Low RK (2003). Intestinal *Oxalobacter formigenes* colonization in calcium oxalate stone formers and its relation to urinary oxalate. J Endourol.

[65] Dawson KA, Allison MJ, Hartman PA (1980). Isolation and some characteristics of anaerobic oxalate-degrading bacteria from the rumen. Appl Environ Microbiol.

[66] Campieri C, Campieri M, Bertuzzi V, Swennen E, Matteuzzi D, Stefoni S (2001). Reduction of oxaluria after an oral course of lactic acid bacteria at high concentration. Kidney Int.

[67] Metges CC, Petzke KJ, El-Khoury AE, Henneman L, Grant I, Bedri S (1999). Incorporation of urea and ammonia nitrogen into ileal and fecal microbial proteins and plasma free amino acids in normal men and ileostomates. Am J Clin Nutr.

[68] Meakins TS, Jackson AA (1996). Salvage of exogenous urea nitrogen enhances nitrogen balance in normal men consuming marginally inadequate protein diets. Clin Sci (London).

[69] Shen T-CD, Albenberg L, Bittinger K, Chehoud C, Chen Y-Y, Judge CA (2015). Engineering the gut microbiota to treat hyperammonemia. J Clin Invest.

[70] Mora D, Arioli S (2014). Microbial urease in health and disease. PLoS Pathog.

[71] Yatsunenko T, Rey FE, Manary MJ, Trehan I, Dominguez-Bello MG, Contreras M (2012). Human gut microbiome viewed across age and geography. Nature.

[72] Pabst O (2012). New concepts in the generation and functions of IgA. Nat Rev Immunol.

[73] Mistry D, Stockley RA (2006). IgA1 protease. Int J Biochem Cell Biol.

[74] Thornton RF, Kagawa TF, O’Toole PW, Cooney JC (2010). The dissemination of C10 cysteine protease genes in *Bacteroides fragilis* by mobile genetic elements. BMC Microbiol.

[75] Thornton RF, Murphy EC, Kagawa TF, O’Toole PW, Cooney JC (2012). The effect of environmental conditions on expression of *Bacteroides fragilis* and *Bacteroides thetaiotaomicron* C10 protease genes. BMC Microbiol.

[76] Potempa M, Potempa J, Kantyka T, Nguyen K-A, Wawrzonek K, Manandhar SP (2009). Interpain A, a cysteine proteinase from *Prevotella intermedia*, inhibits complement by degrading complement factor C3. PLoS Pathog.

[77] Sakamoto M, Lan PT, Benno Y (2007). *Barnesiella viscericola* gen. nov., sp. nov., a novel member of the family *Porphyromonadaceae* isolated from chicken caecum. Int J Syst Evol Microbiol.

[78] Shkoporov AN, Khokhlova EV, Chaplin AV, Kafarskaia LI, Nikolin AA, Polyakov VY (2013). *Coprobacter fastidiosus* gen. nov., sp. nov., a novel member of the family *Porphyromonadaceae* isolated from infant faeces. Int J Syst Evol Microbiol.

[79] Hofstad T, Olsen I, Eribe ER, Falsen E, Collins MD, Lawson PA (2000). *Dysgonomonas* gen. nov. to accommodate *Dysgonomonas gadei* sp. nov., an organism isolated from a human gall bladder, and *Dysgonomonas capnocytophagoides* (formerly CDC group DF-3). Int J Syst Evol Microbiol.

[80] Chen S, Dong X (2005). *Proteiniphilum acetatigenes* gen. nov., sp. nov., from a UASB reactor treating brewery wastewater. Int J Syst Evol Microbiol.

[81] Iino T, Mori K, Itoh T, Kudo T, Suzuki K-i, Ohkuma M (2014). Description of *Mariniphaga anaerophila* gen. nov., sp. nov., a facultatively aerobic marine bacterium isolated from tidal flat sediment, reclassification of the *Draconibacteriaceae* as a later heterotypic synonym of the *Prolixibacteraceae* and description of the family *Marinifilaceae* fam. nov. Int J Syst Evol Microbiol.

[82] Ueki A, Akasaka H, Suzuki D, Ueki K (2006). *Paludibacter propionicigenes* gen. nov., sp. nov., a novel strictly anaerobic, Gram-negative, propionate-producing bacterium isolated from plant residue in irrigated rice-field soil in Japan. Int J Syst Evol Microbiol.

[83] Sakamoto M, Suzuki M, Umeda M, Ishikawa I, Benno Y (2002). Reclassification of *Bacteroides forsythus* (Tanner *et al.* 1986) as *Tannerella forsythensis* corrig., gen. nov., comb. nov. Int J Syst Evol Microbiol.

[84] Sakamoto M, Benno Y (2006). Reclassification of *Bacteroides distasonis*, *Bacteroides goldsteinii* and *Bacteroides merdae* as *Parabacteroides distasonis* gen. nov., comb. nov., *Parabacteroides goldsteinii* comb. nov. and *Parabacteroides merdae* comb. nov. Int J Syst Evol Microbiol.

[85] Soo RM, Skennerton CT, Sekiguchi Y, Imelfort M, Paech SJ, Dennis PG (2014). An expanded genomic representation of the phylum cyanobacteria. Genome Biol Evol.

[86] Li H, Durbin R (2009). Fast and accurate short read alignment with Burrows-Wheeler transform. Bioinformatics.

[87] Bolger AM, Lohse M, Usadel B (2014). Trimmomatic: a flexible trimmer for Illumina sequence data. Bioinformatics.

[88] Paulino D, Warren RL, Vandervalk BP, Raymond A, Jackman SD, Birol I (2015). Sealer: a scalable gap-closing application for finishing draft genomes. BMC Bioinformatics.

[89] Imelfort M, Parks D, Woodcroft BJ, Dennis P, Hugenholtz P, Tyson GW (2014). GroopM: an automated tool for the recovery of population genomes from related metagenomes. Peer J.

[90] Kang DD, Froula J, Egan R, Wang Z. MetaBAT, an efficient tool for accurately reconstructing single genomes from complex microbial communities. In: PeerJ*.* Edited by Rahmann S, vol. 3; 2015: e1165. 10.7717/peerj.1165 PMC4556158 26336640

[91] Parks DH, Imelfort M, Skennerton CT, Hugenholtz P, Tyson GW (2015). CheckM: assessing the quality of microbial genomes recovered from isolates, single cells, and metagenomes. Genome Res.

[92] Katoh K, Standley DM (2013). MAFFT multiple sequence alignment software version 7: improvements in performance and usability. Mol Biol Evol.

[93] Price MN, Dehal PS, Arkin AP (2010). FastTree 2—approximately maximum-likelihood trees for large alignments. PLoS One.

[94] Altschul SF, Gish W, Miller W, Myers EW, Lipman DJ (1990). Basic local alignment search tool. J Mol Biol.

[95] Markowitz VM, Chen I-MA, Palaniappan K, Chu K, Szeto E, Pillay M (2014). IMG 4 version of the integrated microbial genomes comparative analysis system. Nucleic Acids Res.

[96] Kembel SW, Cowan PD, Helmus MR, Cornwell WK, Morlon H, Ackerly DD (2010). Picante: R tools for integrating phylogenies and ecology. Bioinformatics.

[97] Ludwig W, Strunk O, Westram R, Richter L, Meier H, Yadhukumar (2004). ARB: a software environment for sequence data. Nucleic Acids Res.

[98] Seemann T (2014). Prokka: rapid prokaryotic genome annotation. Bioinformatics.

[99] Lechner M, Findeiß S, Steiner L, Marz M, Stadler PF, Prohaska SJ (2011). Proteinortho: detection of (Co-)orthologs in large-scale analysis. BMC Bioinformatics.

[100] Finn RD, Bateman A, Clements J, Coggill P, Eberhardt RY, Eddy SR (2014). Pfam: the protein families database. Nucleic Acids Res.

[101] Eddy SR (2009). A new generation of homology search tools based on probabilistic inference. Genome Inform.

[102] Gibson MK, Forsberg KJ, Dantas G (2015). Improved annotation of antibiotic resistance determinants reveals microbial resistomes cluster by ecology. ISME J.

[103] Moriya Y, Itoh M, Okuda S, Yoshizawa AC, Kanehisa M (2007). KAAS: an automatic genome annotation and pathway reconstruction server. Nucleic Acids Res.

[104] Aziz RK, Bartels D, Best AA, DeJongh M, Disz T, Edwards RA (2008). The RAST server: rapid annotations using subsystems technology. BMC Genomics.

[105] Karp PD, Paley SM, Krummenacker M, Latendresse M, Dale JM, Lee TJ (2010). Pathway Tools version 13.0: integrated software for pathway/genome informatics and systems biology. Brief Bioinform.

[106] Wu M, McNulty NP, Rodionov DA, Khoroshkin MS, Griffin NW, Cheng J et al. Genetic determinants of in vivo fitness and diet responsiveness in multiple human gut *Bacteroides*. Science*.* 2015;350(6256). doi:10.1126/science.aac5992. 10.1126/science.aac5992 PMC4608238 26430127

[107] Yin Y, Mao X, Yang J, Chen X, Mao F, Xu Y (2012). dbCAN: a web resource for automated carbohydrate-active enzyme annotation. Nucleic Acids Res.

[108] Juncker AS, Willenbrock H, Von Heijne G, Brunak S, Nielsen H, Krogh A (2003). Prediction of lipoprotein signal peptides in Gram-negative bacteria. Protein Sci.

[109] Love MI, Huber W, Anders S (2014). Moderated estimation of fold change and dispersion for RNA-seq data with DESeq2. Genome Biol.

[110] Kolde R. pheatmap: Pretty Heatmaps. 2015. R package version 107 from http://CRAN.R-project.org/package=pheatmap.

[111] Roberts D. Labdsv: ordination and multivariate analysis for ecology. 2007. R package version 18-0 from https://cran.r-project.org/web/packages/labdsv/index.html.

[112] Oksanen J, Blanchet FG, Kindt R, Legendre P, Minchin PR, O’Hara RB et al. Vegan: community ecology package. 2015. R package version 23-1 from http://CRAN.R-project.org/package=vegan.

