# Supplementary material for: Genomic characterization of the uncultured Bacteroidales family S24-7 inhabiting the guts of homeothermic animals
Source: Microbiome. 2016 Jul 7;4:36. doi: 10.1186/s40168-016-0181-2 (PMC4936053; doi:10.1186/s40168-016-0181-2)
Supplement: Additional file 1: Table S1. — “Ca. Homeothermaceae” population genome properties. (DOCX 19 kb) [file 40168_2016_181_MOESM1_ESM.docx]

**Table S1. ‘*Ca.* Homeothermaceae’ population genome properties.**

| **Host** | **Genome ID** | **Scaffolds** | **Genome size (Mb)** | **GC (%)** | **CDS** | **CDS density** | **Completeness (%)** | **Contamination (%)** | **Abundance***  **(%)** |
| --- | --- | --- | --- | --- | --- | --- | --- | --- | --- |
| **Human** | H1 | 42 | 2.43 | 50.7 | 2,152 | 0.89 | 85.47 | 1.3 | 13.3 |
|  | H2 | 58 | 1.97 | 55.2 | 1,744 | 0.90 | 80.94 | 0.4 | 1.6 |
|  | H3 | 30 | 2.40 | 54.3 | 2,036 | 0.90 | 94.65 | 1.5 | 14.8 |
|  | H4 | 65 | 2.57 | 51.4 | 2,124 | 0.89 | 96.6 | 0.1 | 7.6 |
|  | H5 | 50 | 2.56 | 56.4 | 2,119 | 0.91 | 85.47 | 0.8 | 1.1 |
|  | H6 | 60 | 2.53 | 52.2 | 2,219 | 0.90 | 88.37 | 1.3 | 0.6 |
|  | H7 | 54 | 2.84 | 48.9 | 2,455 | 0.92 | 99.06 | 1.5 | 0.9 |
|  | H8 | 61 | 2.42 | 50 | 2,116 | 0.91 | 94.72 | 1.1 | 0.9 |
|  | H9 | 48 | 2.09 | 50.9 | 1,741 | 0.91 | 82.29 | 0.4 | 1.3 |
|  | H10 | 174 | 2.91 | 48.3 | 2,871 | 0.89 | 85.77 | 2.1 | 0.8 |
| **Mouse** | M1 | 91 | 3.19 | 48.4 | 2,586 | 0.88 | 98.7 | 1.1 | 1.1 |
|  | M2 | 123 | 3.15 | 49.2 | 2,697 | 0.89 | 95.3 | 0.6 | 1.2 |
|  | M3 | 44 | 2.46 | 54.7 | 2,024 | 0.89 | 87.5 | 2.1 | 0.9 |
|  | M4 | 60 | 2.87 | 50.3 | 2,357 | 0.90 | 99.4 | 0.4 | 7.1 |
|  | M5 | 117 | 2.54 | 52.6 | 2,080 | 0.88 | 93.0 | 0.9 | 0.7 |
|  | M6 | 82 | 2.96 | 50.7 | 2,539 | 0.87 | 98.2 | 3.8 | 0.6 |
|  | M7 | 49 | 2.72 | 50.8 | 2,398 | 0.88 | 96.4 | 0.3 | 3.0 |
|  | M8 | 53 | 3.37 | 56 | 2,822 | 0.89 | 99.4 | 1.4 | 1.6 |
|  | M9 | 121 | 2.65 | 49 | 2,364 | 0.88 | 93.4 | 2.2 | 2.1 |
|  | M10 | 111 | 2.29 | 56.1 | 1,791 | 0.86 | 89.9 | 0.8 | 0.4 |
|  | M11 | 116 | 3.01 | 53.2 | 2,370 | 0.87 | 91.7 | 5.5 | 0.4 |
|  | M12 | 90 | 2.77 | 52.9 | 2,147 | 0.88 | 89.7 | 4.9 | 0.4 |
|  | M13 | 105 | 2.38 | 52.2 | 1,862 | 0.84 | 87.6 | 0.4 | 0.6 |
|  | M14 | 31 | 2.13 | 52.6 | 1,756 | 0.92 | 93.8 | 0.4 | 0.9 |
| **Guinea pig** | GP1 | 64 | 2.20 | 47.5 | 1,870 | 0.88 | 94.3 | 0.7 | 3.1 |
|  | GP2 | 133 | 2.43 | 47.3 | 2,091 | 0.89 | 95.6 | 0.0 | 3.1 |
|  | GP3 | 238 | 2.89 | 44.6 | 2,131 | 0.85 | 81.5 | 2.1 | 3.2 |
|  | GP4 | 276 | 2.69 | 47.3 | 2,295 | 0.83 | 83.9 | 0.2 | 1.5 |
| **Koala** | K1 | 137 | 4.46 | 51.4 | 3,567 | 0.87 | 96.8 | 0.9 | 14.3 |
|  | K10 | 46 | 2.75 | 51.2 | 2,496 | 0.88 | 98.4 | 1.5 | 1.6 |
|  | Average |  | 2.69 | 51.2 | 2,261 | 0.88 | 91.9 | 1.4 | 3.0 |

* Abundance within relevant dataset based on proportion of reads mapping to each specific bin
