# Supplementary material for: Genomic characterization of the uncultured Bacteroidales family S24-7 inhabiting the guts of homeothermic animals
Source: Microbiome. 2016 Jul 7;4:36. doi: 10.1186/s40168-016-0181-2 (PMC4936053; doi:10.1186/s40168-016-0181-2)
Supplement: Additional file 2: Table S2. — Unique genes within each “Ca. Homeothermaceae” population genome. (DOCX 14 kb) [file 40168_2016_181_MOESM2_ESM.docx]

**Table S2. Unique genes within each ‘*Ca.* Homeothermaceae’ population genome.**

| **Genome ID** | **Unique Genes** | **Percentage of total** |
| --- | --- | --- |
| H1 | 212 | 10% |
| H2 | 246 | 14% |
| H3 | 201 | 10% |
| H4 | 376 | 18% |
| H5 | 146 | 7% |
| H6 | 188 | 8% |
| H7 | 246 | 10% |
| H8 | 206 | 10% |
| H9 | 102 | 6% |
| H10 | 765 | 27% |
| M1 | 357 | 14% |
| M2 | 490 | 18% |
| M3 | 270 | 13% |
| M4 | 266 | 11% |
| M5 | 270 | 13% |
| M6 | 529 | 21% |
| M7 | 332 | 14% |
| M8 | 447 | 16% |
| M9 | 502 | 21% |
| M10 | 197 | 11% |
| M11 | 368 | 16% |
| M12 | 203 | 9% |
| M13 | 199 | 11% |
| M14 | 185 | 11% |
| GP1 | 319 | 17% |
| GP2 | 237 | 11% |
| GP3 | 299 | 14% |
| GP4 | 445 | 19% |
| K1 | 1010 | 28% |
| K10 | 458 | 18% |
