# Supplementary figures and images for: Genomic characterization of the uncultured Bacteroidales family S24-7 inhabiting the guts of homeothermic animals
Source: Microbiome. 2016 Jul 7;4:36. doi: 10.1186/s40168-016-0181-2 (PMC4936053; doi:10.1186/s40168-016-0181-2)

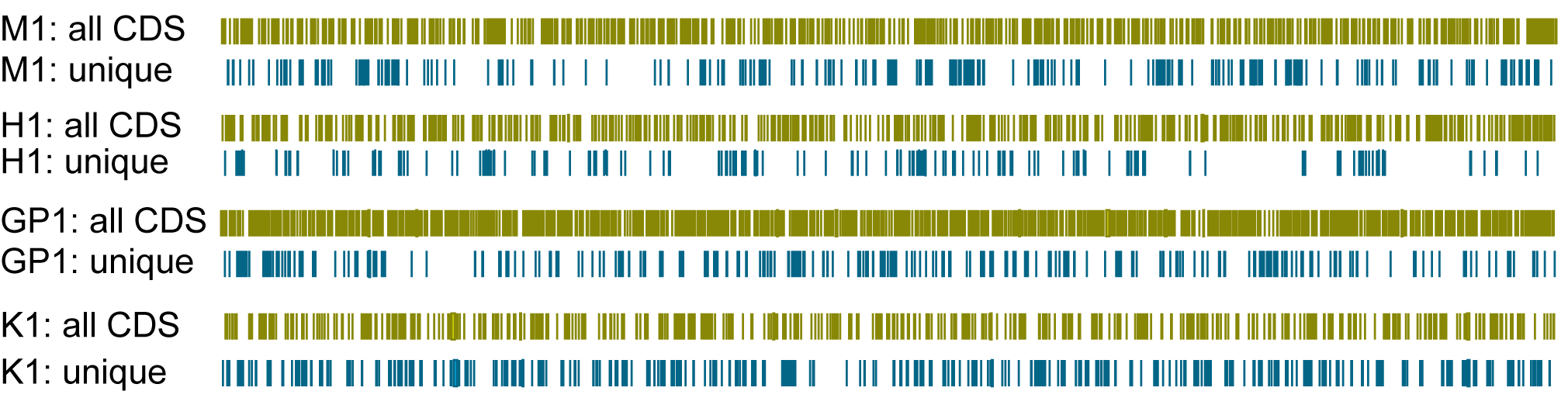

Supplement: Additional file 3: Figure S1. — Distribution of unique genes in selected “Ca. Homeothermaceae” population genomes. Coding sequences within each genome are denoted by vertical yellow lines, unique genes are denoted by vertical blue lines. Classification of unique genes is based on failure to find an ortholog to a given gene in any of the other “Ca. Homeothermaceae” genomes using Proteinortho [99]. (TIF 249 kb) [file 40168_2016_181_MOESM3_ESM.tif]

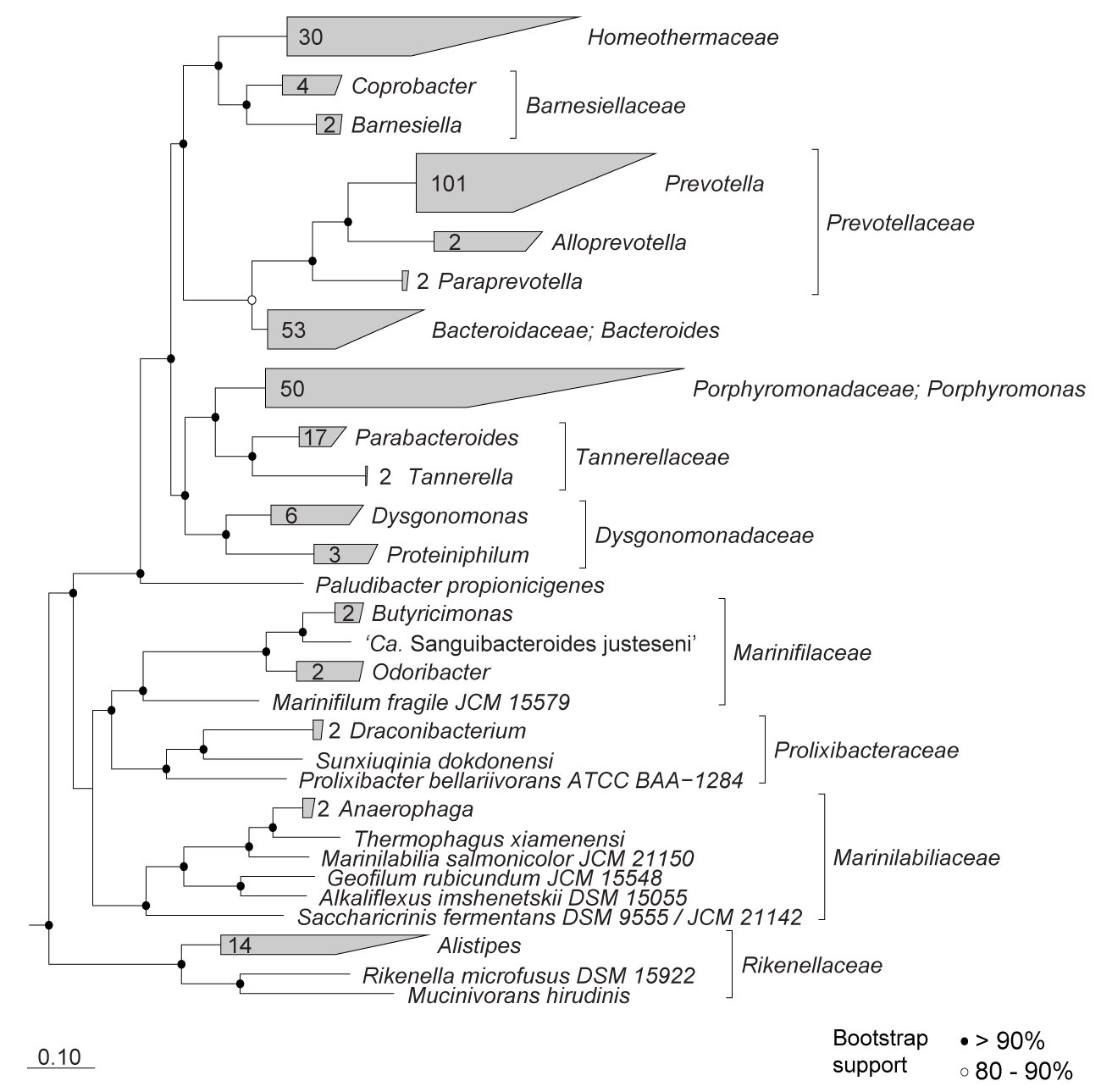

Supplement: Additional file 4: Figure S2. — Phylogenetic tree of the order Bacteroidales. Maximum-likelihood tree of the Bacteroidales based on the concatenated alignment of 120 concatenated marker genes (36,713 amino acids) using genomes available within the NCBI database. Bootstrap support derived from 100 replicates. (TIF 281 kb) [file 40168_2016_181_MOESM4_ESM.tif]

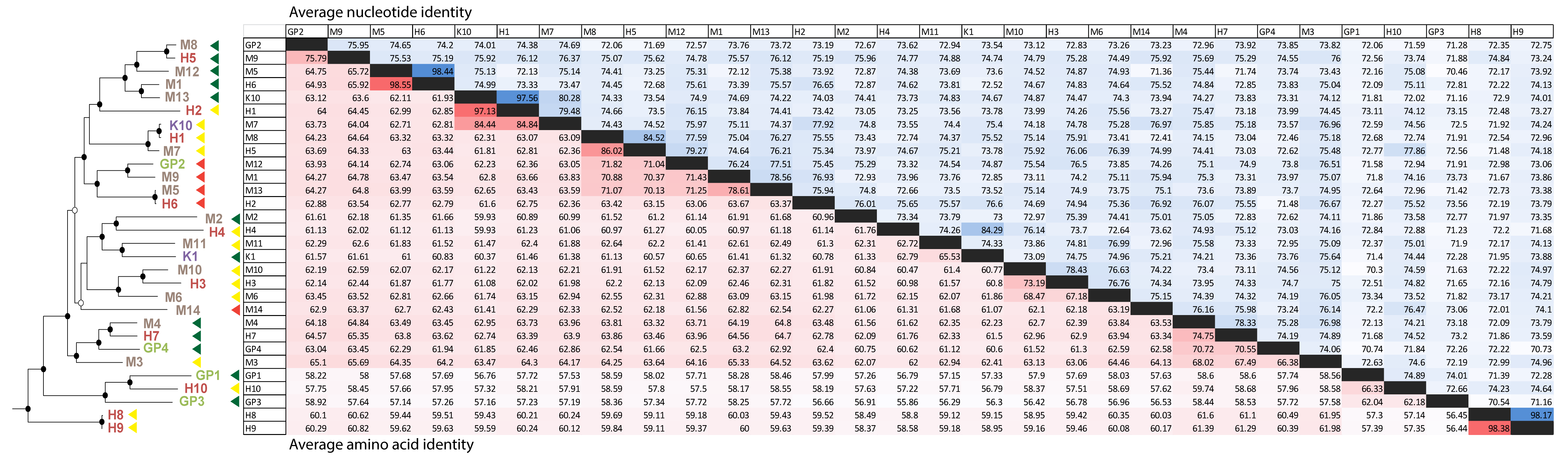

Supplement: Additional file 5: Figure S3. — Average nucleotide and amino acid identity between “Ca. Homeothermaceae” population genomes. ANI calculated using Goris method [18] implemented in calculate_ani.py (https://github.com/widdowquinn/scripts/blob/master/bioinformatics/calculate_ani.py). AAI calculated using CompareM v0.0.5 (https://github.com/dparks1134/CompareM). Values over 95 % indicate population genomes originating from the same species. (TIF 2541 kb) [file 40168_2016_181_MOESM5_ESM.tif]

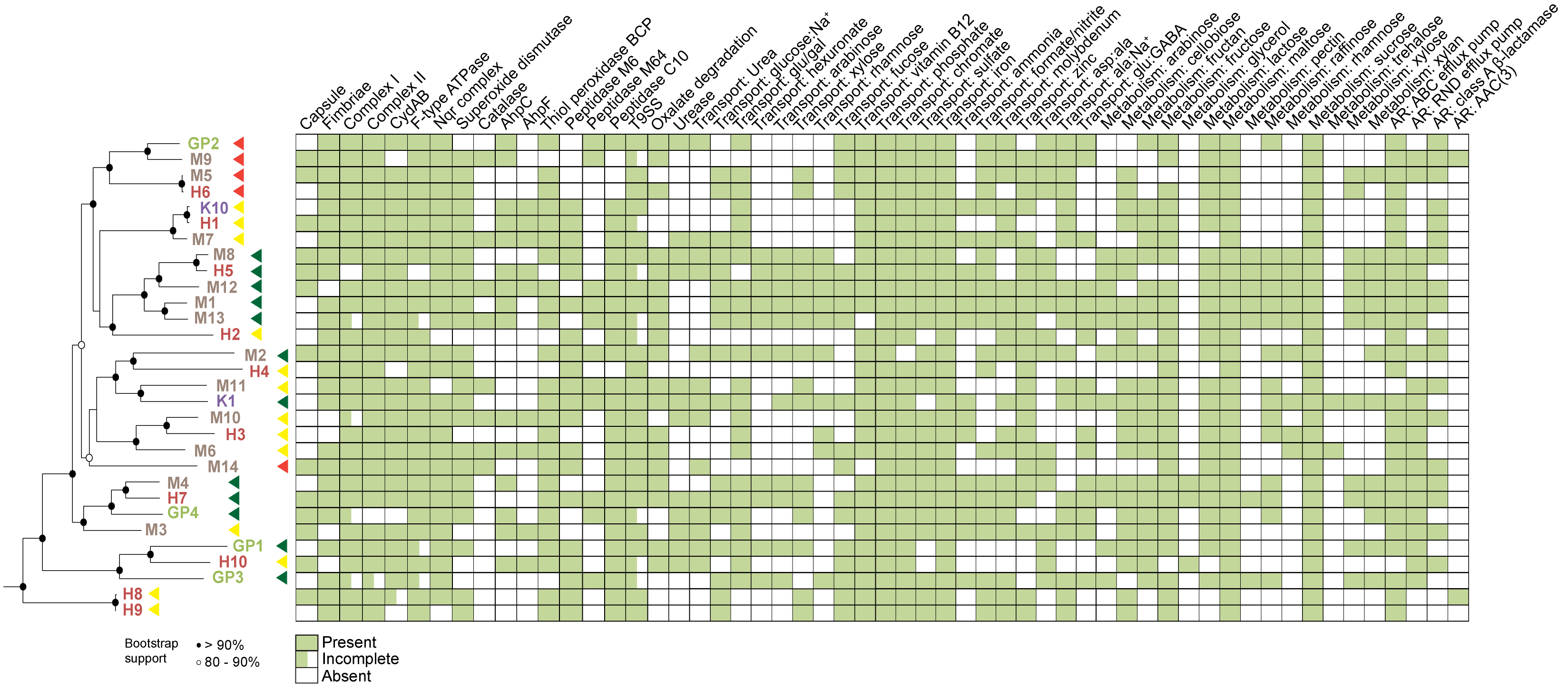

Supplement: Additional file 7: Figure S4. — Presence of described characteristics within “Ca. Homeothermaceae” population genomes. Presence determined via BLAST [94] search of annotated proteins within each population genome. AR: antibiotic resistance. (TIF 724 kb) [file 40168_2016_181_MOESM7_ESM.tif]

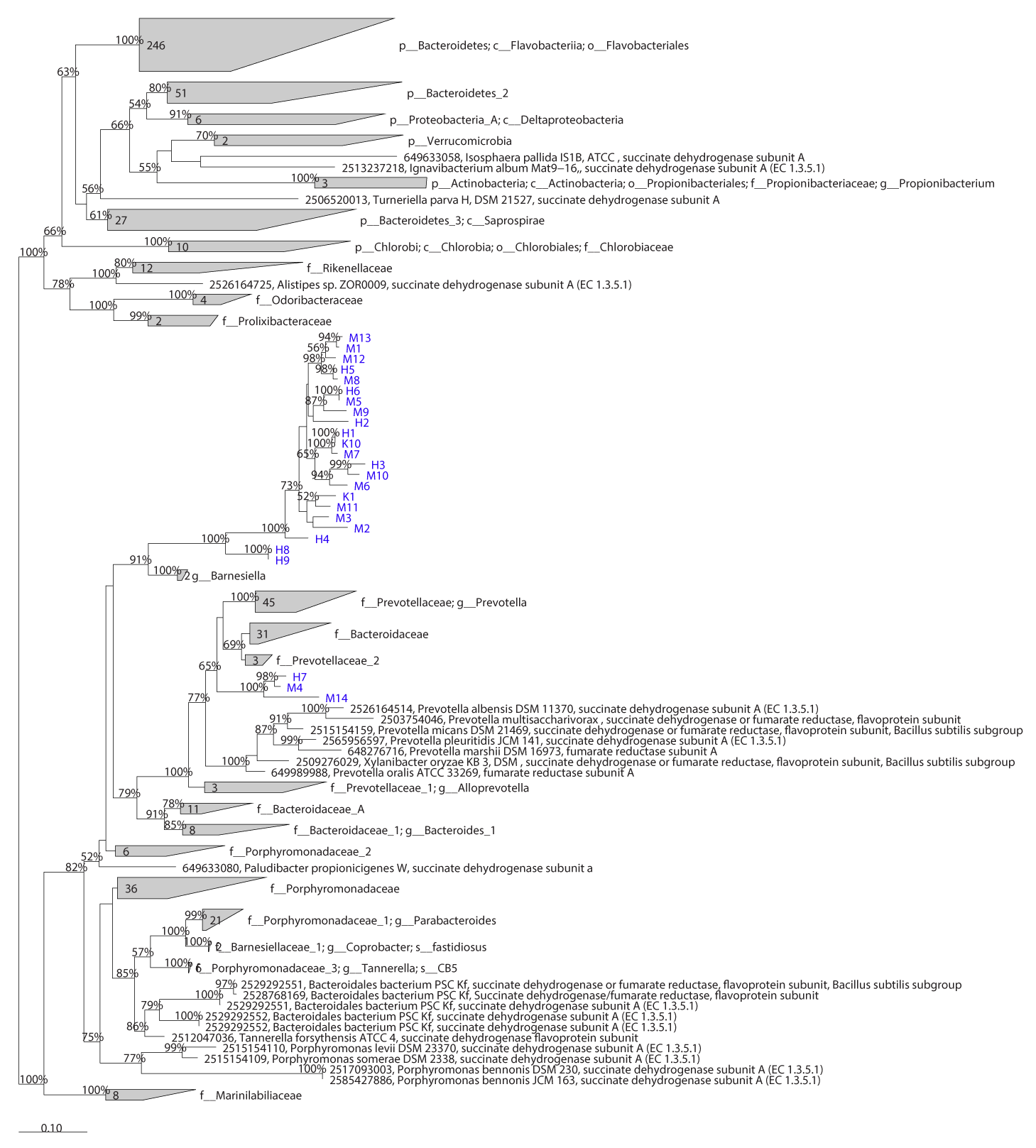

Supplement: Additional file 8: Figure S5. — Gene tree of frdA, catalytic subunit of complex II. Maximum-likelihood gene tree was inferred using FastTree 2 [93] based on a 640 amino acid alignment of sequences, implemented within the in-house script Mingle (https://github.com/Ecogenomics/mingle). Bootstrap values represent result of 100 replicates. “Ca. Homeothermaceae” frdA genes shown in blue. Where shown, tips display IMG genome ID, species name and gene annotation. (TIF 475 kb) [file 40168_2016_181_MOESM8_ESM.tif]

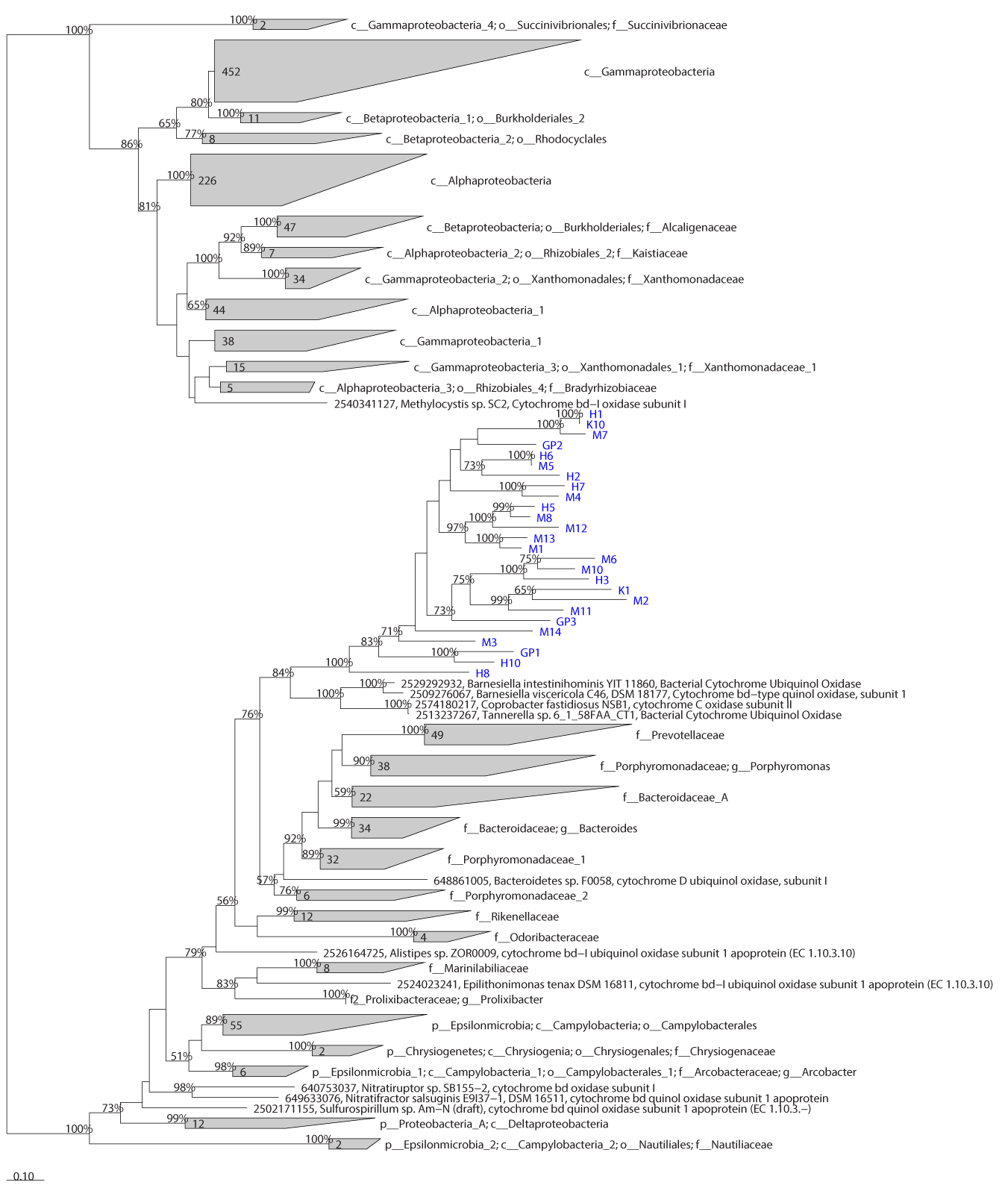

Supplement: Additional file 9: Figure S6. — Gene tree of cydA, cytochrome bd subunit. Maximum-likelihood gene tree was inferred using FastTree 2 [93] based on a 500 amino acid alignment of sequences, implemented within the in-house script Mingle (https://github.com/Ecogenomics/mingle). Bootstrap values represent result of 100 replicates. “Ca. Homeothermaceae” cydA genes shown in blue. Where shown, tips display IMG genome ID, species name and gene annotation. (TIF 347 kb) [file 40168_2016_181_MOESM9_ESM.tif]

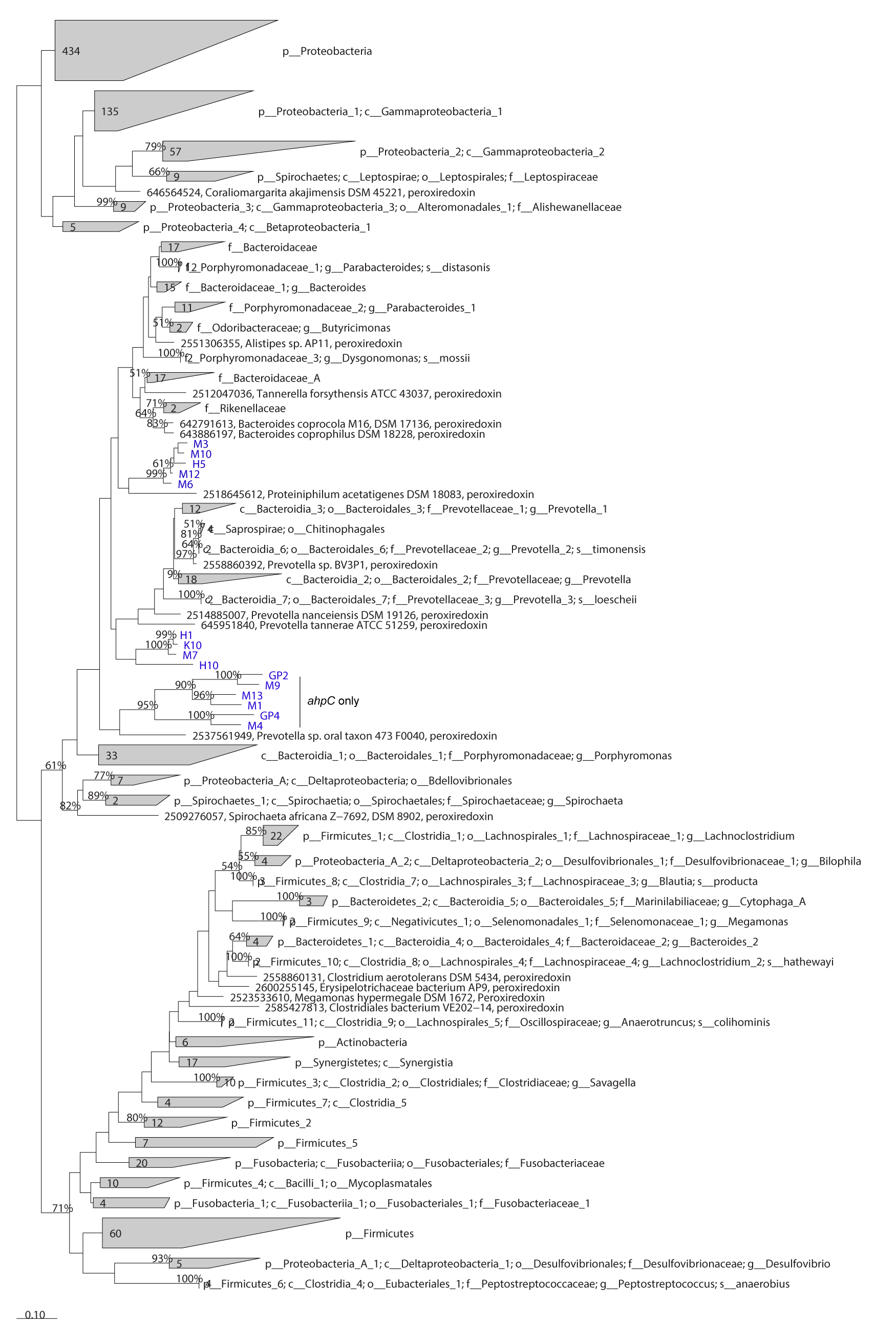

Supplement: Additional file 10: Figure S7. — Gene tree of ahpC, alkyl hydroperoxide. Maximum-likelihood gene tree was inferred using FastTree 2 [93] based on a 180 amino acid alignment of sequences implemented, within the in-house script Mingle (https://github.com/Ecogenomics/mingle). Bootstrap values represent result of 100 replicates. “Ca. Homeothermaceae” ahpC genes shown in blue. Where shown, tips display IMG genome ID, species name and gene annotation. (TIF 787 kb) [file 40168_2016_181_MOESM10_ESM.tif]

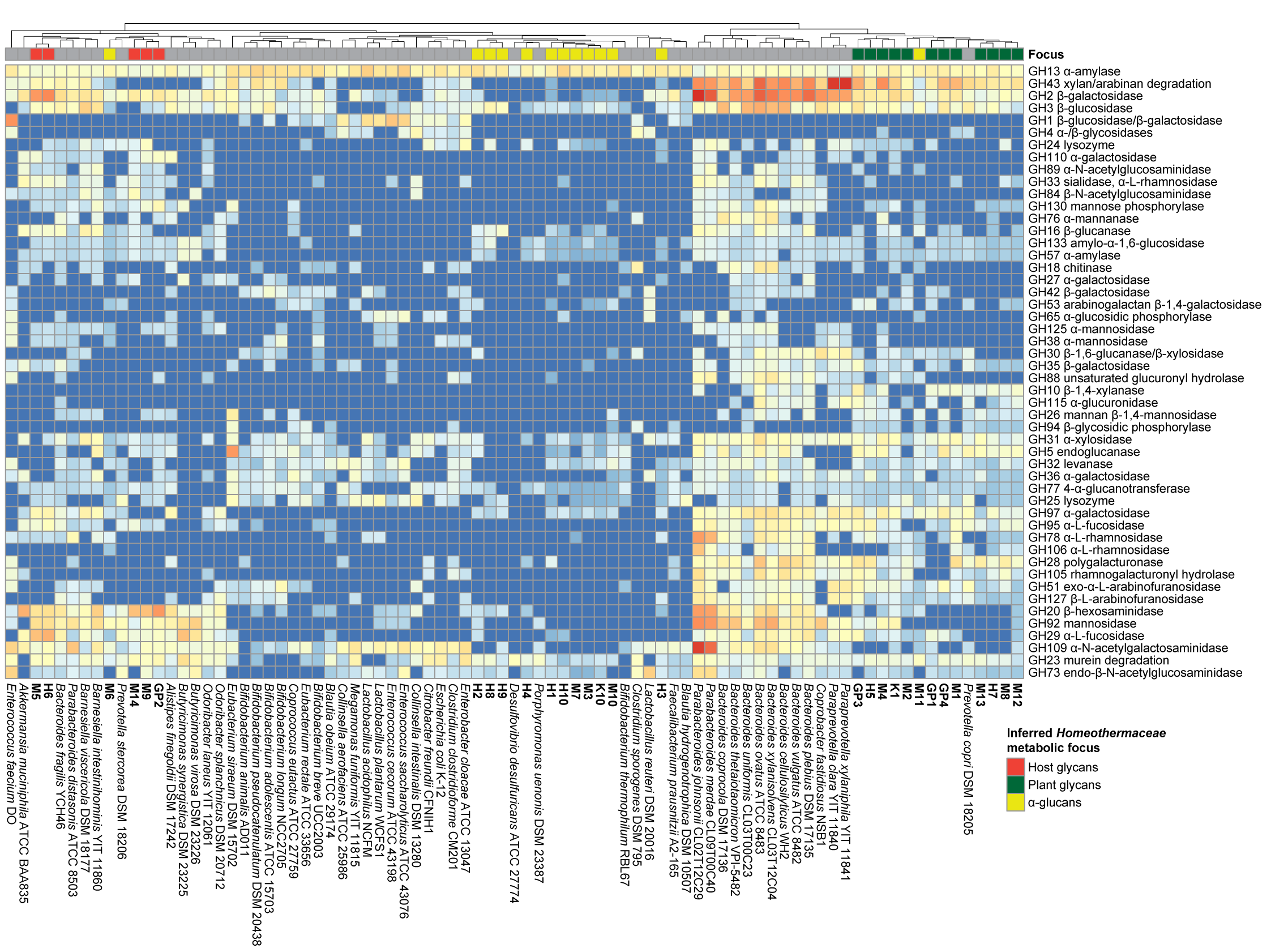

Supplement: Additional file 15: Figure S8. — 50 most abundant glycoside hydrolases in “Ca. Homeothermaceae” and selected gut-associated species. Heatmap displaying the top 50 most abundant GH enzymes within “Ca. Homeothermaceae” in addition to a selection of reference genomes available on NCBI also originating from fecal samples. (TIF 1691 kb) [file 40168_2016_181_MOESM15_ESM.tif]

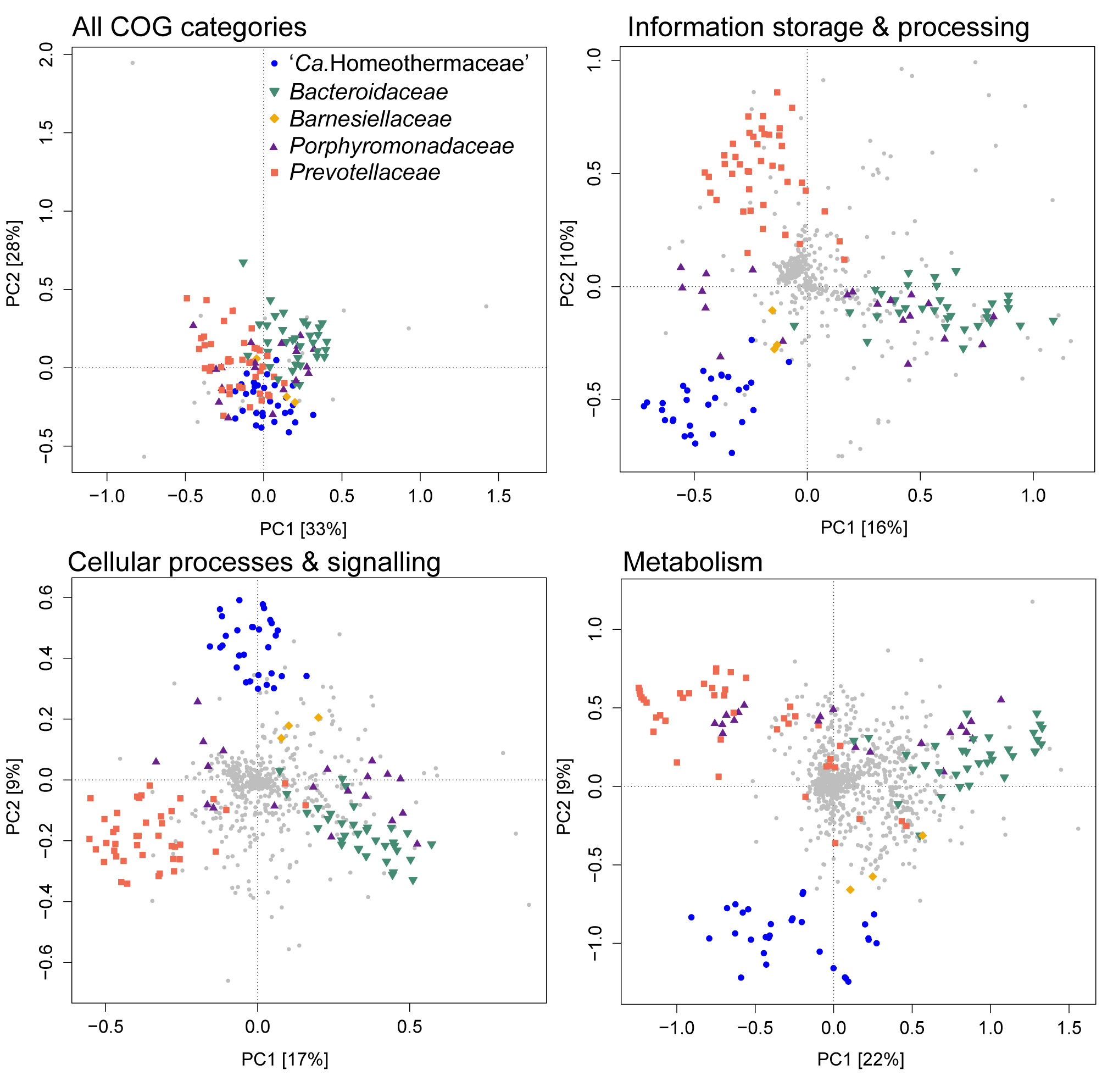

Supplement: Additional file 18: Figure S9. — COG category abundance between “Ca. Homeothermaceae” and related Bacteroidales families. PCA plots generated using annotated COGs within “Ca. Homeothermaceae” and IMG genomes [95] from other families. Family designation of each genome is based on IMG phylogenetic annotation except for Barnesiellaceae, which includes Barnesiella and Coprobacter (previously Porphyromonadaceae). (TIF 550 kb) [file 40168_2016_181_MOESM18_ESM.tif]

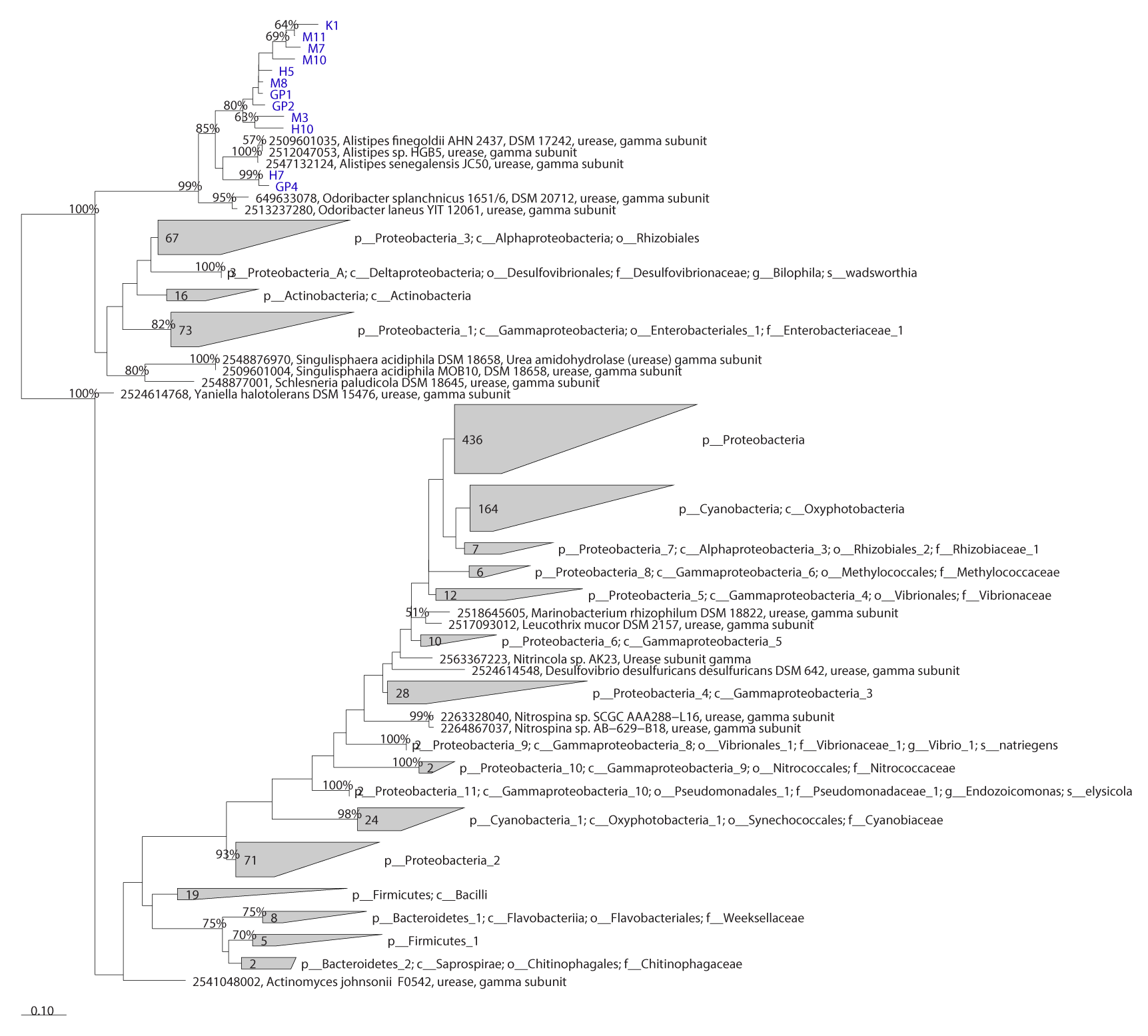

Supplement: Additional file 20: Figure S10. — Gene tree of ureA, urease subunit. Maximum-likelihood gene tree was inferred using FastTree 2 [93] based on a 100 amino acid alignment of sequences, implemented within the in-house script Mingle (https://github.com/Ecogenomics/mingle). Bootstrap values represent result of 100 replicates. “Ca. Homeothermaceae” ureA genes shown in blue. Where shown, tips display IMG genome ID, species name and gene annotation. (TIF 470 kb) [file 40168_2016_181_MOESM20_ESM.tif]

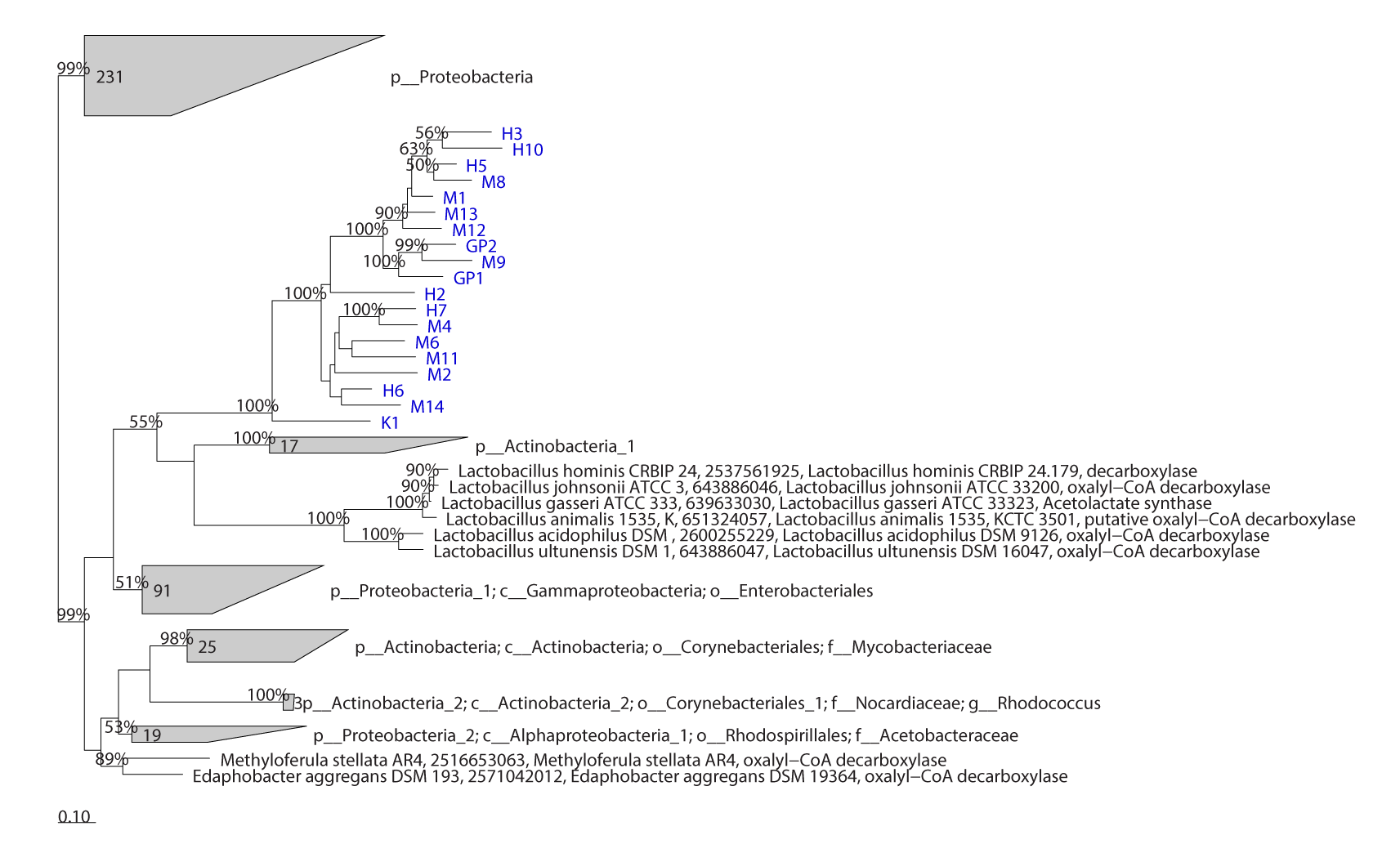

Supplement: Additional file 21: Figure S11. — Gene tree of oxa, oxalyl-CoA decarboxylase. Maximum-likelihood gene tree was inferred using FastTree 2 [93] based on a 580 amino acid alignment of sequences, implemented within the in-house script Mingle (https://github.com/Ecogenomics/mingle). Bootstrap values represent result of 100 replicates. “Ca. Homeothermaceae” oxa genes shown in blue. Where shown, tips display IMG genome ID, species name and gene annotation. (TIF 296 kb) [file 40168_2016_181_MOESM21_ESM.tif]
