# Supplementary material for: Genomic characterization of the uncultured Bacteroidales family S24-7 inhabiting the guts of homeothermic animals
Source: Microbiome. 2016 Jul 7;4:36. doi: 10.1186/s40168-016-0181-2 (PMC4936053; doi:10.1186/s40168-016-0181-2)
Supplement: Additional file 11: Table S4. — Top 20 most abundant CAZy categories within “Ca. Homeothermaceae” as a percentage of total genes with CAZy annotation in each genome. (DOCX 17 kb) [file 40168_2016_181_MOESM11_ESM.docx]

**Table S4.** **Top 20 most abundant CAZy categories within ‘*Ca.* Homeothermaceae’ as a percentage of total genes with CAZy annotation** **in each genome.**

| **Category** | **‘*Ca.* Homeothermaceae’** | **Human average** | **Mouse average** | **Guinea pig average** | **Koala average** | ***Porphyromonadaceae*** | ***Prevotellaceae*** | ***Bacteroidaceae*** | **Mouse gut metagenome** | **Soil metagenome** | **Marine metagenome** |
| --- | --- | --- | --- | --- | --- | --- | --- | --- | --- | --- | --- |
| **GT2** | 14.0% | 14.0% | 15.0% | 8.9% | 16.4% | 11.2% | 13.7% | 8.8% | 14.3% | 10.5% | 10.5% |
| **GT4** | 8.3% | 8.5% | 9.1% | 5.1% | 8.1% | 8.0% | 5.3% | 6.4% | 7.4% | 9.8% | 11.3% |
| **GH13** | 6.4% | 8.5% | 5.4% | 4.1% | 7.9% | 2.0% | 3.6% | 1.9% | 4.5% | 3.7% | 2.1% |
| **GH43** | 3.1% | 1.6% | 3.3% | 5.8% | 3.7% | 1.8% | 2.4% | 5.2% | 2.4% | 0.3% | 0.5% |
| **CBM50** | 2.3% | 2.7% | 1.7% | 3.1% | 2.4% | 5.2% | 2.9% | 2.3% | 3.7% | 1.6% | 2.9% |
| **CE1** | 3.5% | 3.2% | 2.6% | 6.7% | 4.6% | 4.1% | 3.9% | 2.5% | 2.1% | 5.2% | 5.1% |
| **GH2** | 3.0% | 2.2% | 3.2% | 3.7% | 3.6% | 3.4% | 2.7% | 5.7% | 2.5% | 0.2% | 0.3% |
| **CE10** | 2.9% | 2.8% | 2.9% | 2.9% | 3.0% | 3.2% | 2.4% | 1.8% | 1.9% | 4.0% | 3.4% |
| **CBM32** | 2.0% | 1.4% | 2.5% | 1.8% | 1.4% | 3.4% | 3.3% | 4.6% | 2.3% | 0.8% | 0.5% |
| **CBM26** | 1.7% | 2.2% | 1.6% | 0.0% | 2.5% | 0.0% | 0.3% | 0.1% | 0.3% | 0.0% | 0.0% |
| **GH3** | 2.6% | 3.3% | 1.9% | 2.9% | 3.7% | 1.9% | 1.9% | 3.2% | 2.9% | 3.0% | 2.2% |
| **GH23** | 2.1% | 2.4% | 2.1% | 1.8% | 2.2% | 1.9% | 1.3% | 1.0% | 1.0% | 3.2% | 2.8% |
| **GH97** | 1.9% | 1.8% | 1.8% | 2.9% | 2.2% | 1.0% | 1.4% | 2.1% | 0.4% | 0.0% | 0.1% |
| **CBM48** | 2.1% | 2.6% | 1.8% | 2.1% | 2.2% | 0.9% | 1.0% | 1.0% | 1.4% | 1.8% | 0.9% |
| **GH20** | 1.5% | 1.0% | 1.8% | 2.3% | 0.5% | 1.8% | 1.7% | 2.5% | 0.8% | 0.3% | 0.3% |
| **GH31** | 1.6% | 1.4% | 1.6% | 2.1% | 1.8% | 0.6% | 0.8% | 1.3% | 1.1% | 0.3% | 0.1% |
| **GH5** | 1.5% | 1.0% | 1.6% | 2.3% | 1.1% | 0.4% | 0.9% | 1.0% | 0.9% | 0.6% | 0.5% |
| **CBM20** | 1.1% | 1.3% | 1.0% | 1.0% | 1.3% | 0.9% | 1.3% | 0.6% | 0.2% | 0.1% | 0.2% |
| **CE3** | 1.5% | 1.5% | 1.5% | 1.4% | 1.3% | 1.7% | 1.3% | 1.9% | 1.1% | 0.9% | 0.7% |
| **GT5** | 1.5% | 1.6% | 1.5% | 1.4% | 1.3% | 1.3% | 1.3% | 0.7% | 0.8% | 0.4% | 0.5% |
| **Total CAZy genes / total CDS** | 6.3% | 5.2% | 6.4% | 6.3% | 5.4% | 5.3% | 5.7% | 7.5% | 3.8% | 1.0% | 1.2% |
