# Supplementary material for: Genomic characterization of the uncultured Bacteroidales family S24-7 inhabiting the guts of homeothermic animals
Source: Microbiome. 2016 Jul 7;4:36. doi: 10.1186/s40168-016-0181-2 (PMC4936053; doi:10.1186/s40168-016-0181-2)
Supplement: Additional file 12: Table S5. — CAZy categories with significantly different abundance in “Ca. Homeothermaceae” in comparison with collated counts from Bacteroidaceae, Prevotellaceae, and Porphyromonadaceae. (DOCX 17 kb) [file 40168_2016_181_MOESM12_ESM.docx]

**Table S5. CAZy categories with significantly different abundance in ‘*Ca.* Homeothermaceae’ in comparison with collated counts from *Bacteroidacea*e, *Prevotellaceae* and *Porphyromonadaceae.***

| Category | Description | *P*-value | ‘*Ca.* Homeothermaceae’ | *Bacteroidaceaeae* | *Prevotellaceae* | *Porphyromonadaceae* |
| --- | --- | --- | --- | --- | --- | --- |
| GH13 | alpha-amylase; pullulanase; | 1.52E-08 | 6.4% | 1.9% | 3.6% | 2.0% |
| GH109 | alpha-N-acetylgalactosaminidase | 6.33E-08 | 0.7% | 2.2% | 1.9% | 2.6% |
| CBM26 | Starch-binding function demonstrated | 1.37E-06 | 1.7% | 0.1% | 0.3% | 0.0% |
| GH92 | mannosyl-oligosaccharide alpha-1,2-mannosidase; | 2.95E-05 | 1.1% | 3.1% | 1.9% | 3.3% |
| PL8 | hyaluronate lyase; chondroitin AC lyase; xanthan lyase; | 0.0002 | 0.0% | 0.6% | 0.1% | 0.2% |
| GH18 | chitinase; endo-beta-N-acetylglucosaminidase; | 0.0004 | 0.0% | 1.0% | 0.6% | 0.2% |
| GH88 | d-4,5 unsaturated beta-glucuronyl hydrolase | 0.0004 | 0.2% | 1.1% | 0.2% | 0.3% |
| GT51 | murein polymerase | 0.0004 | 1.0% | 1.0% | 2.0% | 1.4% |
| CBM32 | Binding to galactose and lactose has been demonstrated | 0.0004 | 2.0% | 4.6% | 3.3% | 3.4% |
| GH63 | alpha-glucosidase; alpha-1,3-glucosidase; alpha-glucosidase | 0.0008 | 0.0% | 0.5% | 0.1% | 0.1% |
| CBM9 | cellulose-binding function within xylanases | 0.0037 | 0.1% | 0.5% | 0.3% | 0.4% |
| GH33 | sialidase or neuraminidase; trans-sialidase; | 0.0102 | 0.3% | 0.9% | 0.6% | 0.7% |
| GH76 | alpha-1,6-mannanase | 0.0105 | 0.2% | 1.0% | 0.4% | 0.3% |
| GH125 | exo-alpha-1,6-mannosidase | 0.0167 | 0.2% | 0.5% | 0.4% | 0.4% |
| GH130 | 1-β-D-mannopyranosyl-4-D-glucopyranose; β-1,4-mannooligosaccharide phosphorylase | 0.0167 | 0.5% | 1.0% | 0.6% | 0.6% |
| CBM67 | L-rhamnose binding activity | 0.0167 | 0.4% | 1.3% | 0.7% | 0.7% |
| CBM48 | glycogen-binding function, appended to GH13 modules | 0.0266 | 2.1% | 1.0% | 1.0% | 0.9% |
| CE9 | N-acetylglucosamine 6-phosphate deacetylase; | 0.0290 | 0.1% | 0.6% | 0.1% | 0.3% |
| GH2 | beta-galactosidase; beta-mannosidase; | 0.0308 | 3.0% | 5.7% | 2.7% | 3.4% |
| GH27 | alpha-galactosidase; alpha-N-acetylgalactosaminidase; | 0.0308 | 0.1% | 0.3% | 0.4% | 0.2% |
| GH127 | β-L-arabinofuranosidase | 0.0308 | 0.4% | 1.0% | 0.5% | 0.5% |
| CBM4 | Binding demonstrated with xylan, beta-1,3-glucan, | 0.0308 | 0.7% | 0.3% | 0.3% | 0.2% |
| CBM66 | Targets the terminal fructoside residue of fructans | 0.0308 | 0.1% | 0.5% | 0.1% | 0.5% |
| GH89 | alpha-N-acetylglucosaminidase | 0.0363 | 0.2% | 0.5% | 0.3% | 0.2% |
| PL12 | heparin-sulfate lyase | 0.0363 | 0.3% | 0.8% | 0.2% | 0.2% |
| GH94 | cellobiose phosphorylase; cellodextrin phosphorylase; | 0.0400 | 0.2% | 0.0% | 0.1% | 0.0% |
| GH123 | glycosphingolipid beta-N-acetylgalactosaminidase | 0.0400 | 0.0% | 0.4% | 0.1% | 0.2% |
| GH29 | alpha-L-fucosidase | 0.0416 | 1.1% | 1.7% | 1.2% | 1.5% |
| CBM35 | binding xylan and mannose demonstrated | 0.0457 | 1.0% | 0.4% | 0.2% | 0.3% |
