# Supplementary material for: Genomic characterization of the uncultured Bacteroidales family S24-7 inhabiting the guts of homeothermic animals
Source: Microbiome. 2016 Jul 7;4:36. doi: 10.1186/s40168-016-0181-2 (PMC4936053; doi:10.1186/s40168-016-0181-2)
Supplement: Additional file 13: Table S6. — Significantly enriched carbohydrate-active enzymes within each trophic guild. (DOCX 18 kb) [file 40168_2016_181_MOESM13_ESM.docx]

**Table S6. Significantly enriched carbohydrate active enzymes within each trophic guild.**

| **Significantly enriched enzymes within α-glucan guild** | | | | | | |
| --- | --- | --- | --- | --- | --- | --- |
|  | ***P*-value versus** | |  | **Enzyme count per guild** | | |
|  | **Host** | **Plant** |  | **α-glucan** | **Plant** | **Host** |
| GH13 | 0.0002 | 0.0016 | alpha-amylase; pullulanase; cyclomaltodextrin glucanotransferase *etc.* | 144 | 78 | 10 |
| CBM26 | 0.0056 | 7.21E-05 | Starch-binding function demonstrated in two cases. | 54 | 2 | 0 |
|  |  |  |  |  |  |  |
| **Significantly enriched enzymes within plant guild** | | | | | | |
|  | ***P*-value versus** | |  | **Enzyme count per guild** | | |
|  | **Host** | **α-glucan** |  | **α-glucan** | **Plant** | **Host** |
| GH43 | 0.0002 | 1.65E-12 | beta-xylosidase; beta-1,3-xylosidase; alpha-L-arabinofuranosidase *etc.* | 5 | 142 | 7 |
| GH28 | 0.0097 | 0.0002 | polygalacturonase; exo-polygalacturonase; exo-polygalacturonosidase *etc.* | 7 | 57 | 0 |
| GH10 | 0.0140 | 4.57E-05 | endo-1,4-beta-xylanase; endo-1,3-beta-xylanase | 0 | 34 | 0 |
| GH51 | 0.0140 | 0.0003 | alpha-L-arabinofuranosidase; endoglucanase | 1 | 32 | 0 |
| GH105 | 0.0140 | 7.20E-05 | unsaturated rhamnogalacturonyl hydrolase | 0 | 29 | 0 |
| PL1 | 0.0140 | 0.0001 | pectate lyase; exo-pectate lyase; pectin lyase | 0 | 38 | 0 |
| CE8 | 0.0150 | 0.0007 | pectin methylesterase | 1 | 39 | 0 |
| CBM4 | 0.0287 | 0.0059 | Binding demonstrated with xylan, beta-1,3-glucan, beta-1,3-1,4-glucan, beta-1,6-glucan and amorphous cellulose but not with crystalline cellulose. | 5 | 27 | 0 |
| PL11 | 0.0304 | 0.0004 | rhamnogalacturonan lyase; exo-unsaturated rhamnogalacturonan lyase | 0 | 19 | 0 |
| CE12 | 0.0304 | 0.0035 | pectin acetylesterase; rhamnogalacturonan acetylesterase; acetyl xylan esterase | 2 | 23 | 0 |
| GH5 | 0.0426 | 0.0021 | chitosanase; beta-mannosidase; cellulase *etc.* | 14 | 48 | 3 |
| GH115 | 0.0426 | 0.0007 | xylan alpha-1,2-glucuronidase; alpha-(4-O-methyl)-glucuronidase | 0 | 16 | 0 |
|  |  |  |  |  |  |  |
| **Significantly enriched enzymes within host glycan guild** | | | | | | |
|  | ***P*-value versus** | |  | **Enzyme count per guild** | | |
|  | **Plant** | **α-glucan** |  | **α-glucan** | **Plant** | **Host** |
| GH20 | 1.10E-08 | 1.05E-08 | beta-hexosaminidase; lacto-N-biosidase; beta-1,6-N-acetylglucosaminidase *etc.* | 10 | 6 | 38 |
| CBM32 | 0.0005 | 3.28E-05 | Binding to galactose and lactose has been demonstrated | 17 | 22 | 39 |
| GH84 | 0.0042 | 0.0140 | N-acetyl beta-glucosaminidase; hyaluronidase | 1 | 0 | 7 |
| GH29 | 0.0140 | 3.28E-05 | alpha-L-fucosidase | 5 | 17 | 24 |
| GH33 | 0.0426 | 0.0052 | sialidase or neuraminidase; trans-sialidase; 2-keto-3-deoxynononic acid sialidase | 1 | 5 | 8 |

Showing enzymes significantly enriched in both pairwise comparisons for each guild. All enzymes were also identified using indicator species analysis.
