# Supplementary material for: Genomic characterization of the uncultured Bacteroidales family S24-7 inhabiting the guts of homeothermic animals
Source: Microbiome. 2016 Jul 7;4:36. doi: 10.1186/s40168-016-0181-2 (PMC4936053; doi:10.1186/s40168-016-0181-2)
Supplement: Additional file 14: Table S7. — Indicator carbohydrate-active enzymes identified within each trophic guild. (DOCX 23 kb) [file 40168_2016_181_MOESM14_ESM.docx]

**Table S7. Indicator carbohydrate active enzymes identified within each trophic guild.**

| **Indicator enzymes identified in α-glucan guild** | | | | | | |
| --- | --- | --- | --- | --- | --- | --- |
|  |  |  |  | **Enzyme count per guild** | | |
|  | **Score** | ***P-*value** | **Description** | **α-glucan** | **Plant** | **Host** |
| **CBM26** | 0.8135 | 0.001 | Starch-binding function demonstrated in two cases. | 54 | 2 | 0 |
| **GH13** | 0.5658 | 0.001 | alpha-amylase; pullulanase; cyclomaltodextrin glucanotransferase *etc.* | 144 | 78 | 10 |
| GH128 | 0.4663 | 0.023 | β-1,3-glucanase | 7 | 1 | 0 |
|  |  |  |  |  |  |  |
| **Indicator enzymes identified in plant guild** | | | | | | |
|  |  |  |  | **Enzyme count per guild** | | |
|  | **Score** | ***P-*value** | **Description** | **α-glucan** | **Plant** | **Host** |
| **GH51** | 0.972 | 0.001 | alpha-L-arabinofuranosidase; endoglucanase | 1 | 32 | 0 |
| **GH43** | 0.869 | 0.001 | beta-xylosidase; beta-1,3-xylosidase; alpha-L-arabinofuranosidase *etc.* | 5 | 142 | 7 |
| GH127 | 0.8599 | 0.001 | ?-L-arabinofuranosidase | 3 | 17 | 0 |
| **GH10** | 0.8333 | 0.001 | endo-1,4-beta-xylanase; endo-1,3-beta-xylanase | 0 | 34 | 0 |
| **GH105** | 0.8333 | 0.001 | unsaturated rhamnogalacturonyl hydrolase | 0 | 29 | 0 |
| **GH28** | 0.7485 | 0.003 | polygalacturonase; exo-polygalacturonase; exo-polygalacturonosidase *etc.* | 7 | 57 | 0 |
| **CBM4** | 0.7117 | 0.001 | Binding demonstrated with xylan, beta-1,3-glucan, beta-1,3-1,4-glucan, beta-1,6-glucan and amorphous cellulose but not with crystalline cellulose. | 5 | 27 | 0 |
| CE6 | 0.7116 | 0.001 | acetyl xylan esterase | 5 | 41 | 5 |
| GH67 | 0.6667 | 0.001 | alpha-glucuronidase; xylan alpha-1,2-glucuronidase | 0 | 8 | 0 |
| **GH115** | 0.6667 | 0.001 | xylan alpha-1,2-glucuronidase; alpha-(4-O-methyl)-glucuronidase | 0 | 16 | 0 |
| **PL11** | 0.6667 | 0.004 | rhamnogalacturonan lyase; exo-unsaturated rhamnogalacturonan lyase | 0 | 19 | 0 |
| GH35 | 0.6635 | 0.002 | beta-galactosidase; exo-beta-glucosaminidase | 1 | 15 | 2 |
| **GH5** | 0.6459 | 0.002 | chitosanase; beta-mannosidase; cellulase *etc.* | 14 | 48 | 3 |
| GH36 | 0.6246 | 0.003 | alpha-galactosidase; alpha-N-acetylgalactosaminidase; stachyose synthase *etc.* | 5 | 15 | 1 |
| GH94 | 0.6223 | 0.003 | cellobiose phosphorylase; cellodextrin phosphorylase; chitobiose phosphorylase *etc.* | 2 | 9 | 0 |
| **CE12** | 0.6171 | 0.007 | pectin acetylesterase; rhamnogalacturonan acetylesterase; acetyl xylan esterase | 2 | 23 | 0 |
| GH31 | 0.6159 | 0.001 | alpha-glucosidase; alpha-1,3-glucosidase; sucrase-isomaltase | 20 | 45 | 4 |
| CE7 | 0.5988 | 0.002 | acetyl xylan esterase; cephalosporin-C deacetylase | 4 | 16 | 2 |
| **PL1** | 0.5833 | 0.009 | pectate lyase; exo-pectate lyase; pectin lyase | 0 | 38 | 0 |
| CBM6 | 0.5715 | 0.009 | The cellulose-binding function has been demonstrated in one case on amorphous cellulose and beta-1,4-xylan | 3 | 27 | 4 |
| **CE8** | 0.5698 | 0.003 | pectin methylesterase | 1 | 39 | 0 |
| CE2 | 0.5657 | 0.011 | acetyl xylan esterase | 6 | 17 | 0 |
| GH95 | 0.554 | 0.01 | alpha-1,2-L-fucosidase; alpha-L-fucosidase | 0 | 33 | 9 |
| GH97 | 0.5468 | 0.004 | alpha-glucosidase; alpha-galactosidase | 25 | 51 | 8 |
| GH78 | 0.5331 | 0.018 | alpha-L-rhamnosidase | 7 | 20 | 2 |
| PL10 | 0.5 | 0.006 | pectate lyase | 0 | 6 | 0 |
| GH3 | 0.4949 | 0.009 | beta-glucosidase; xylan 1,4-beta-xylosidase; beta-N-acetylhexosaminidase *etc.* | 40 | 55 | 8 |
| GH26 | 0.4764 | 0.026 | beta-mannanase; beta-1,3-xylanase | 6 | 18 | 2 |
| CBM48 | 0.4596 | 0.007 | Glycogen-binding function, appended to GH13 modules | 38 | 38 | 4 |
| CE10 | 0.4457 | 0.014 | arylesterase; carboxyl esterase; acetylcholinesterase *etc.* | 38 | 61 | 17 |
| GH106 | 0.4281 | 0.027 | alpha-L-rhamnosidase | 2 | 11 | 0 |
| GH30 | 0.3991 | 0.046 | glucosylceramidase; beta-1,6-glucanase; beta-xylosidase | 5 | 10 | 0 |
| GH8 | 0.3333 | 0.024 | chitosanase; cellulase; licheninase *etc.* | 0 | 5 | 0 |
| GH42 | 0.3333 | 0.036 | beta-galactosidase | 0 | 5 | 0 |
| PL4 | 0.3333 | 0.031 | rhamnogalacturonan lyase | 0 | 4 | 0 |
| CBM16 | 0.3333 | 0.025 | Binding to cellulose and glucomannan demonstrated | 0 | 4 | 0 |
| CBM61 | 0.3333 | 0.021 | A beta-1,4-galactan binding function has been demonstrated | 0 | 4 | 0 |
|  |  |  |  |  |  |  |
| **Indicator species identified in host glycan guild** | | | | | | |
|  |  |  |  | **Enzyme count per guild** | | |
|  | **Score** | ***P-*value** | **Description** | **α-glucan** | **Plant** | **Host** |
| **GH84** | 0.9479 | 0.001 | N-acetyl beta-glucosaminidase; hyaluronidase | 1 | 0 | 7 |
| GH110 | 0.8679 | 0.001 | alpha-galactosidase; alpha-1,3-galactosidase | 1 | 2 | 8 |
| **GH20** | 0.8569 | 0.001 | beta-hexosaminidase; lacto-N-biosidase; beta-1,6-N-acetylglucosaminidase *etc.* | 10 | 6 | 38 |
| GH89 | 0.8041 | 0.002 | alpha-N-acetylglucosaminidase | 1 | 2 | 5 |
| **GH33** | 0.7642 | 0.001 | sialidase or neuraminidase; trans-sialidase; 2-keto-3-deoxynononic acid sialidase | 1 | 5 | 8 |
| **GH29** | 0.7271 | 0.002 | alpha-L-fucosidase | 5 | 17 | 24 |
| **CBM32** | 0.7129 | 0.001 | Binding to galactose and lactose has been demonstrated | 17 | 22 | 39 |
| GH125 | 0.6665 | 0.001 | exo-alpha-1,6-mannosidase | 1 | 1 | 4 |
| GH109 | 0.6661 | 0.001 | alpha-N-acetylgalactosaminidase | 10 | 4 | 11 |
| GH92 | 0.6453 | 0.001 | mannosyl-oligosaccharide alpha-1,2-mannosidase; mannosyl-oligosaccharide alpha-1,3-mannosidase | 8 | 19 | 20 |
| GH130 | 0.6215 | 0.007 | 1-?-D-mannopyranosyl-4-D-glucopyranose:phosphate ?-D-mannosyltransferase; ?-1,4-mannooligosaccharide phosphorylase *etc.* | 4 | 8 | 8 |
| CBM51 | 0.6 | 0.003 | Binding to galactose and to blood group A/B-antigens demonstrated | 0 | 0 | 3 |
| CE14 | 0.5843 | 0.008 | N-acetyl-1-D-myo-inosityl-2-amino-2-deoxy-alpha-D-glucopyranoside deacetylase (EC 3.5.1.89); diacetylchitobiose deacetylase (EC 3.5.1.-); mycothiol S-conjugate amidase (EC 3.5.1.-) | 6 | 3 | 5 |
| CBM13 | 0.4735 | 0.026 | Binding to xylan has been demonstrated | 1 | 1 | 3 |
| CBM62 | 0.4481 | 0.021 | Binding to galactose has been demonstrated | 6 | 2 | 4 |
| GH120 | 0.4 | 0.03 | beta-xylosidase | 0 | 0 | 4 |
| GH123 | 0.4 | 0.022 | glycosphingolipid beta-N-acetylgalactosaminidase | 0 | 0 | 2 |
| CBM12 | 0.4 | 0.018 | The majority of these modules is found among chitinases where the function is chitin-binding | 0 | 0 | 2 |
| GT21 | 0.3656 | 0.05 | UDP-Glc: ceramide beta-glucosyltransferase | 5 | 0 | 3 |
| CE9 | 0.3355 | 0.039 | N-acetylglucosamine 6-phosphate deacetylase; N-acetylgalactosamine-6-phosphate deacetylase | 1 | 0 | 2 |

Bold enzymes also detected as enriched in both pairwise comparisons using DESeq2
