# Supplementary material for: Genomic characterization of the uncultured Bacteroidales family S24-7 inhabiting the guts of homeothermic animals
Source: Microbiome. 2016 Jul 7;4:36. doi: 10.1186/s40168-016-0181-2 (PMC4936053; doi:10.1186/s40168-016-0181-2)
Supplement: Additional file 16: Table S8. — SusCD-like genes present in “Ca. Homeothermaceae” genomes. (DOCX 17 kb) [file 40168_2016_181_MOESM16_ESM.docx]

**Table S8. *SusCD*-like genes present in ‘*Ca.* Homeothermaceae’ genomes**

| **Genome** | ***susC*-like genes** | ***susD*-like genes** | **ECF-σ factor genes** | **HTCS genes** | | ***susCD*-like pairs** | **Pairs with CAZymes** | **Pairs with CAZymes plus sensor** |
| --- | --- | --- | --- | --- | --- | --- | --- | --- |
| **H1** | 6 | 8 | 1 | 2 | | 7 | 6 | 2 |
| **H2** | 4 | 4 | 1 | 0 | | 4 | 2 | 0 |
| **H3** | 3 | 4 | 0 | 2 | | 4 | 1 | 1 |
| **H4** | 2 | 3 | 0 | 1 | | 3 | 0 | 0 |
| **H5** | 7 | 8 | 2 | 2 | | 8 | 8 | 2 |
| **H6** | 4 | 4 | 1 | 0 | | 7 | 1 | 0 |
| **H7** | 11 | 13 | 1 | 5 | | 13 | 10 | 4 |
| **H8** | 6 | 4 | 0 | 1 | | 4 | 1 | 0 |
| **H9** | 3 | 5 | 0 | 1 | | 4 | 2 | 0 |
| **H10** | 7 | 7 | 0 | 1 | | 7 | 5 | 1 |
| **M1** | 14 | 22 | 2 | 6 | | 23 | 10 | 6 |
| **M2** | 9 | 10 | 3 | 4 | | 11 | 7 | 4 |
| **M3** | 9 | 10 | 2 | 1 | | 10 | 5 | 1 |
| **M4** | 16 | 17 | 4 | 7 | | 16 | 11 | 6 |
| **M5** | 6 | 6 | 1 | 0 | | 10 | 0 | 0 |
| **M6** | 6 | 10 | 2 | 3 | | 11 | 6 | 1 |
| **M7** | 4 | 5 | 0 | 1 | | 5 | 2 | 1 |
| **M8** | 6 | 12 | 2 | 3 | | 12 | 10 | 3 |
| **M9** | 5 | 9 | 1 | 0 | | 12 | 6 | 0 |
| **M10** | 6 | 8 | 0 | 3 | | 7 | 5 | 2 |
| **M11** | 18 | 18 | 6 | 3 | | 18 | 10 | 0 |
| **M12** | 14 | 26 | 4 | 5 | | 24 | 11 | 4 |
| **M13** | 12 | 14 | 2 | 3 | | 14 | 7 | 3 |
| **M14** | 2 | 2 | 1 | 0 | | 3 | 2 | 0 |
| **GP1** | 3 | 5 | 2 | 2 | | 5 | 3 | 2 |
| **GP2** | 11 | 13 | 2 | 2 | | 11 | 4 | 2 |
| **GP3** | 15 | 24 | 2 | 7 | | 20 | 10 | 2 |
| **GP4** | 8 | 14 | 3 | 8 | | 14 | 7 | 4 |
| **K1** | 27 | 30 | 3 | 11 | | 30 | 20 | 8 |
| **K10** | 6 | 8 | 1 | 5 | | 7 | 5 | 2 |
| **Average per trophic guild** | | | | |  | | | |
| **Plant glycan** | 12 | 16 | 2 | 5 | | 16 | 10 | 4 |
| **α-glucan** | 6 | 7 | 1 | 2 | | 7 | 4 | 1 |
| **Host glycan** | 6 | 7 | 1 | 1 | | 9 | 3 | 1 |
