# Supplementary material for: Genomic characterization of the uncultured Bacteroidales family S24-7 inhabiting the guts of homeothermic animals
Source: Microbiome. 2016 Jul 7;4:36. doi: 10.1186/s40168-016-0181-2 (PMC4936053; doi:10.1186/s40168-016-0181-2)
Supplement: Additional file 17: Table S9. — Significantly enriched COGs within each trophic guild. (DOCX 15 kb) [file 40168_2016_181_MOESM17_ESM.docx]

**Table S9. Significantly enriched COGs within each trophic guild**

| **Significantly enriched COGs within α-glucan guild** | | | | | | |
| --- | --- | --- | --- | --- | --- | --- |
|  | ***P*-value versus** | |  | **Enzyme count per guild** | | |
|  | **Host** | **Plant** |  | **α-glucan** | **Plant** | **Host** |
| COG0366 | 0.0001 | 0.0017 | Glycosidase | 101 | 34 | 5 |
| **Significantly enriched COGs within plant guild** | | | | | | |
|  | ***P*-value versus** | |  | **Enzyme count per guild** | | |
|  | **Host** | **α-glucan** |  | **α-glucan** | **Plant** | **Host** |
| COG3507 | 2.37E-06 | 2.42E-14 | Beta-xylosidase | 3 | 139 | 6 |
| COG3693 | 0.0004 | 8.63E-06 | Endo-1,4-beta-xylanase, GH35 family | 0 | 38 | 0 |
| COG4677 | 0.0004 | 0.0001 | Pectin methylesterase and related acyl-CoA thioesterases | 3 | 43 | 0 |
| COG2730 | 0.0013 | 0.0282 | Aryl-phospho-beta-D-glucosidase BglC, GH1 family | 10 | 38 | 0 |
| COG3866 | 0.0162 | 0.0001 | Pectate lyase | 0 | 37 | 1 |
| **Significantly enriched COGs within host glycan guild** | | | | | | |
|  | ***P*-value versus** | |  | **Enzyme count per guild** | | |
|  | **Plant** | **α-glucan** |  | **α-glucan** | **Plant** | **Host** |
| COG3525 | 3.93E-09 | 6.78E-08 | N-acetyl-beta-hexosaminidase | 12 | 7 | 49 |
| COG3119 | 0.0013 | 2.05E-05 | Arylsulfatase A or related enzyme | 8 | 14 | 30 |
| COG3669 | 0.0327 | 1.45E-05 | Alpha-L-fucosidase | 5 | 19 | 30 |

Bold enzymes also detected as enriched in both pairwise comparisons using DESeq2
