# Supplementary material for: Genomic characterization of the uncultured Bacteroidales family S24-7 inhabiting the guts of homeothermic animals
Source: Microbiome. 2016 Jul 7;4:36. doi: 10.1186/s40168-016-0181-2 (PMC4936053; doi:10.1186/s40168-016-0181-2)
Supplement: Additional file 22: Table S11. — Prevalence and relative abundance of “Ca. Homeothermaceae” populations within public metagenomics datasets from human and mouse gut. (DOCX 25 kb) [file 40168_2016_181_MOESM22_ESM.docx]

**Table S11. Prevalence and relative abundance of ‘*Ca.* Homeothermaceae’ populations within public human and mouse fecal metagenomic datasets.**

|  | | | **Prevalence and abundance in murine datasets** | | | | | | **Prevalence and abundance in human datasets** | | | | | | | |
| --- | --- | --- | --- | --- | --- | --- | --- | --- | --- | --- | --- | --- | --- | --- | --- | --- |
| **Population genome** | **Guild** | | **Overall prevalence**  **(n = 100)** | **Overall average abundance (n = 100)** | **SC diet prevalence**  **(n = 69)** | **SC diet average abundance (n = 69)** | **HF diet prevalence**  **(n = 31)** | **HF diet average abundance (n = 31)** | **Overall prevalence**  **(n = 307)** | **Overall average abundance (n = 307)** | **Hadza prevalence**  **(n = 27)** | **Hadza average abundance (n = 27)** | **Lean prevalence**  **(n = 29)** | **Lean average abundance (n = 29)** | **Obese prevalence**  **(n = 31)** | **Obese average abundance (n = 31)** |
| **H1** | α-glucan | |  |  |  |  |  |  | 1.6% | 2.6% |  |  |  |  | 3.2% | 0.5% |
| **H2** | α-glucan | |  |  |  |  |  |  | 0.7% | 1.1% |  |  |  |  |  |  |
| **H3** | α-glucan | | 3.0% | 0.7% | 4.6% | 0.7% |  |  | 2.0% | 3.1% |  |  | 6.9% | 1.0% | 3.2% | 0.6% |
| **H4** | α-glucan | |  |  |  |  |  |  | 5.9% | 1.3% | 55.6% | 0.9% |  |  |  |  |
| **H5** | Plant glycan | |  |  |  |  |  |  | 0.3% | 1.1% |  |  |  |  |  |  |
| **H6** | Host glycan | |  |  |  |  |  |  | 0.3% | 0.5% |  |  |  |  |  |  |
| **H7** | Plant glycan | |  |  |  |  |  |  | 0.3% | 0.9% |  |  |  |  |  |  |
| **H8** | α-glucan | |  |  |  |  |  |  | 13.0% | 0.9% | 29.6% | 0.7% | 3.4% | 0.8% | 16.1% | 1.6% |
| **H9** | α-glucan | |  |  |  |  |  |  | 11.7% | 0.8% | 44.4% | 0.7% | 3.4% | 0.6% | 16.1% | 1.0% |
| **H10** | α-glucan | | 1.0% | 0.6% | 1.5% | 0.6% |  |  | 0.3% | 0.8% |  |  |  |  |  |  |
| **M1** | Plant glycan | | 4.0% | 1.1% | 5.8% | 1.1% |  |  |  |  |  |  |  |  |  |  |
| **M2** | Plant glycan | |  |  |  |  |  |  |  |  |  |  |  |  |  |  |
| **M3** | α-glucan | |  |  |  |  |  |  |  |  |  |  |  |  |  |  |
| **M4** | Plant glycan | | 4.0% | 1.4% | 4.4% | 1.6% | 3.2% | 0.7% |  |  |  |  |  |  |  |  |
| **M5** | Host glycan | |  |  |  |  |  |  |  |  |  |  |  |  |  |  |
| **M6** | α-gluc. | Host | 35.0% | 3.5% | 39.1% | 3.8% | 25.8% | 2.6% |  |  |  |  |  |  |  |  |
| **M7** | α-glucan | |  |  |  |  |  |  |  |  |  |  |  |  |  |  |
| **M8** | Plant glycan | |  |  |  |  |  |  |  |  |  |  |  |  |  |  |
| **M9** | Host glycan | | 12.0% | 0.6% | 17.4% | 0.6% |  |  | 0.3% | 0.5% |  |  |  |  |  |  |
| **M10** | α-glucan | | 4.0% | 3.6% | 5.8% | 3.6% |  |  |  |  |  |  |  |  |  |  |
| **M11** | α-gluc. | Plant |  |  |  |  |  |  |  |  |  |  |  |  |  |  |
| **M12** | Plant glycan | | 1.0% | 0.5% | 1.5% | 0.5% |  |  |  |  |  |  |  |  |  |  |
| **M13** | Plant glycan | | 4.0% | 0.6% | 4.4% | 0.6% | 3.2% | 0.6% |  |  |  |  |  |  |  |  |
| **M14** | Host glycan | | 6.0% | 1.1% | 5.8% | 1.3% | 6.5% | 0.6% |  |  |  |  |  |  |  |  |
| **GP1** | Plant glycan | |  |  |  |  |  |  |  |  |  |  |  |  |  |  |
| **GP2** | Host glycan | |  |  |  |  |  |  |  |  |  |  |  |  |  |  |
| **GP3** | Plant glycan | |  |  |  |  |  |  |  |  |  |  |  |  |  |  |
| **GP4** | Plant glycan | |  |  |  |  |  |  |  |  |  |  |  |  |  |  |
| **K1** | Plant glycan | |  |  |  |  |  |  |  |  |  |  |  |  |  |  |
| **K10** | α-glucan | |  |  |  |  |  |  | 0.3% | 3.0% |  |  |  |  |  |  |
| **Total prevalence*** |  | | 47% |  | 54% |  | 32% |  | 20% |  | 70% |  | 10% |  | 23% |  |
| **Average relative abundance^#^** |  | |  | 6.1% |  | 7.1% |  | 3.6% |  | 1.9% |  | 1.5% |  | 1.1% |  | 1.9% |

*Count of presence of one or more populations within a single sample

^#^Average relative abundance based on samples where ‘*Ca*. Homeothermaceae’ populations were present in detectable abundance (≥0.5%)

SC: standard chow; HF: high-fat chow
